# Patient-specific prostate tumour growth simulation: a first step towards the digital twin

**DOI:** 10.3389/fphys.2024.1421591

**Published:** 2024-10-30

**Authors:** Ángela Pérez-Benito, José Manuel García-Aznar, María José Gómez-Benito, María Ángeles Pérez

**Affiliations:** Multiscale in Mechanical and Biological Engineering (M2BE), Aragon Institute of Engineering Research (I3A), University of Zaragoza, Zaragoza, Spain

**Keywords:** prostate cancer, *in-silico* model, patient-specific, imaging biomarkers, computational oncology, finite element method (FEM)

## Abstract

Prostate cancer (PCa) is a major world-wide health concern. Current diagnostic methods involve Prostate-Specific Antigen (PSA) blood tests, biopsies, and Magnetic Resonance Imaging (MRI) to assess cancer aggressiveness and guide treatment decisions. MRI aligns with *in silico* medicine, as patient-specific image biomarkers can be obtained, contributing towards the development of digital twins for clinical practice. This work presents a novel framework to create a personalized PCa model by integrating clinical MRI data, such as the prostate and tumour geometry, the initial distribution of cells and the vasculature, so a full representation of the whole prostate is obtained. On top of the personalized model construction, our approach simulates and predicts temporal tumour growth in the prostate through the Finite Element Method, coupling the dynamics of tumour growth and the transport of oxygen, and incorporating cellular processes such as proliferation, differentiation, and apoptosis. In addition, our approach includes the simulation of the PSA dynamics, which allows to evaluate tumour growth through the PSA patient’s levels. To obtain the model parameters, a multi-objective optimization process is performed to adjust the best parameters for two patients simultaneously. This framework is validated by means of data from four patients with several MRI follow-ups. The diagnosis MRI allows the model creation and initialization, while subsequent MRI-based data provide additional information to validate computational predictions. The model predicts prostate and tumour volumes growth, along with serum PSA levels. This work represents a preliminary step towards the creation of digital twins for PCa patients, providing personalized insights into tumour growth.

## 1 Introduction

Prostate cancer (PCa) is a major health concern world-wide, being the most common cancer and the third leading cause of cancer-related deaths in men after lung and colorectal cancers. Projections indicate that by 2040 in Europe, the incidence rate of PCa is expected to rise by 27.6%, with mortality increasing by 53.2%, according to the World Health Organization (WHO).

The routine methods employed for the diagnosis of the PCa generally include blood tests to measure the Prostate-Specific Antigen (PSA) level, digital rectal examinations, transrectal ultrasounds, prostate biopsies, and/or imaging techniques like Magnetic Resonance Imaging (MRI) (Phan et al., 2020; Lorenzo et al., 2016; Visschere, 2018). Clinicians assign a level of aggressiveness of the cancer with these methods and establish the course of treatment to be considered (Rebello et al., 2021). Pathologists assess biopsy samples and assign a primary Gleason Score (GS), which represents the predominant histological pattern, and a secondary grade for the highest observed pattern. Both grades are assigned on a scale ranging from 1 to 5, based on microscopic architectural features and cellular characteristics. Clinicians have traditionally categorized PCa diagnoses into low, intermediate, and high-risk categories, considering a combination of Gleason patterns, PSA levels, and clinical stage (Litwin and Tan, 2017). From MRI, risk is assigned according to the PiRADs v2 protocol based on the textures of the images, the location of the tumour and its volume (Andrés et al., 2017; Weinreb et al., 2016). Specialized image acquisitions techniques can be incorporated in order to help assessing the cancer. Multiparametric MRI (mpMRI) typically includes diffusion-weighted (DWI) and dynamic contrast-enhanced imaging (DCE), in addition to T2-weighted imaging (T2w) (Litwin and Tan, 2017). Upon confirming the presence of a tumour, clinicians select the most appropriate treatment based on the patient’s risk group. Common treatments for PCa include radical prostatectomy, radiotherapy (RT), hormone therapy (HT), and active surveillance (AS). The PSA blood test plays a crucial role in various stages of PCa management, including screening, assessing future risk, detecting recurrent disease after local therapy, and managing advanced disease (Pezaro et al., 2014). AS is activated only in low-risk and some intermediate risk patients.

The integration of advanced medical imaging techniques like MRI is in line with the principles of *in silico* medicine (Rebello et al., 2021), which utilizes personalized digital models to improve disease prevention, diagnosis, prognosis, and treatment. In addition, it is possible to derive quantifiable parameters from these imaging techniques. The Apparent Diffusion Coefficient (ADC), derived from DWI, quantifies the diffusion of water molecules within tissue and it has been shown that it inversely correlates with the tissue cellularity (Atuegwu et al., 2013). Moreover, DCE-MRI sequences are employed to estimate tumour vascularization using pharmacokinetic models. The Standard Tofts Model (STM) can be applied to characterize tissue vascularization, considering two compartments: the extravascular extracellular space $\left(v_{e}\right)$ and the intravascular space $\left(v_{p}\right)$ . The exchange of substances between these compartments is modeled through the $K^{\mathrm{Trans}}$ variable, which represents the extravasation rate and depends on blood flow, vascular surface area, and vascular permeability, thereby providing an overview of vascularization (Langer et al., 2010) that is closely related to oxygen levels and nutrients. Therefore, thanks to these images and different segmentation techniques, it is possible to create digital replicas of the desired organs that incorporate the vascular and cellular characteristics of each individual patient (Chase et al., 2018). This enables the potential development of patient-specific models for clinical uses, incorporating their unique parameters, which significantly enhances the comprehension of the intricate and diverse nature of these diseases (Hassanzadeh et al., 2017; Litwin and Tan, 2017; Epstein et al., 2016; Viceconti, 2015; Chase et al., 2018).

Mathematical modeling in cancer research is a multifaceted field that involves critical decisions on the framework and scale of models, balancing biological accuracy with computational feasibility. Bull and Byrne (2022) identified six key mathematical hallmarks or decisions: single *versus* hybrid frameworks, homogeneity *versus* heterogeneity, spatially averaged *versus* spatially resolved, single-scale *versus* multi-scale, deterministic *versus* stochastic, and continuum *versus* discrete. Different mathematical models of cancer combine these hallmarks in different ways, leading to models that may include more biological complexity but are more challenging to analyze and/or parameterize (Greene et al. (2015); Agosti et al. (2018); Protopapa et al. (2018); Suzuki et al. (2021). For an accurate representation of tumour growth, it is essential to consider the surrounding tumour microenvironment. This includes various factors that may promote or inhibit growth. Modeling these influences often involves transport equations that account for substances like oxygen (Mpekris et al., 2015). Moreover, the model can incorporate various treatment modalities, from traditional methods like chemotherapy to cutting-edge approaches involving nanoparticles, to predict their impact on tumour progression (Dogra et al., 2019). Furthermore, the process of angiogenesis, where new blood vessels form to supply nutrients to growing tumours, is key to modeling substance delivery. Therefore, *in silico* models of tumour-induced angiogenesis are crucial for understanding and optimizing drug delivery. Moreover, angiogenesis models can be informed and tuned using routinely-acquired imaging data, e.g., angiography images, ultrasound, elastography data, or magnetic resonance images of the tumour anatomy (Hadjicharalambous et al., 2021). However, translating these models from research to clinical practice faces significant hurdles, such as parameter specification, mirroring *in-vivo* conditions, and the need for extensive validation data to ensure accuracy and reliability. Overcoming these challenges is essential for these models to enhance cancer treatment and patient outcomes (Hadjicharalambous et al., 2021).

Recent advances in PCa modeling have focused on integrating cutting-edge technologies to improve diagnosis and treatment strategies. Current models aim to describe the complex interactions between tumoral cells and their microenvironment (Phan et al., 2020). A key feature of many of these models is the inclusion of PSA as a primary indicator of PCa progression. For instance, studies by Lorenzo et al. (2016) and Mohammadi et al. (2021) used the phase-field method to account for the dynamics and co-existence of healthy and cancerous cells, as well as their interaction with nutrients, using partial differential equations (PDEs). These models are capable of predicting not only tumour growth but also PSA dynamics. Notably, Lorenzo et al. (2016) incorporated real prostate geometries extracted from CT images, enhancing the model’s personalized accuracy. Efforts have also been made to model treatment responses. Lorenzo and collaborators (2020) extended their previous work to predict tumour shrinkage under HT and Jain et al. (2011) studied the response of cancer cells under intermittent HT to predict treatment failure also taking into account PSA. In addition, PSA response to RT, factoring in tumour population and radiation-induced damage, was modeled by Lorenzo et al. (2019b).

While these works represent significant advances in PCa modeling, they still have the potential to benefit from more comprehensive clinical data obtained from routine practices, such as MRI, biopsies, and biochemical analyses. In a more recent study, Lorenzo et al. (2024) implemented a spatio-temporal mechanistic model informed by patient-specific data, using T2w and DWI MRI biomarkers to characterize tumour growth under AS. Although this is a robust and complex model, it could be further enhanced by incorporating patient-specific parameters from DCE MRI to evaluate prostate vascularization. Therefore, a more accurate representation of PCa can be obtained through mpMRI, being able not only to get an idea of the cellularity, but also of the vascularity of the prostate, a key feature in the development and treatment of this tumour.

In this work, a patient-specific model of prostate tumour growth is proposed, integrating individualised MRI-derived imaging biomarkers with a biomechanical computational model, using the ADC, as a measure of cellularity and the $K^{\mathrm{Trans}}$ , as a parameter that defines vascularization characteristics. This combined and integrative approach aims to enhance the ability to predict tumour growth, understand its interactions within the prostate, and forecast PSA evolution under AS conditions. A multispecies model of partial differential reaction-advection-diffusion equations coupled with the mechanics of continuous media is here presented. Additionally, we included the dynamics of PSA with cancer development. This may help to establish a connection between patient-specific PSA levels and the aggressiveness of the cancer. In this way, the growth of the tumour can be predicted and therefore help to determine the best possible stage to apply an alternative treatment in case it is necessary. This work represents a preliminary step of a PCa model that in a future may contribute to the creation of a digital twin for PCa. The approach presented here derived from a previous tumour growth model created for neuroblastoma developed by Hervas-Raluy et al. (2024).

## 2 Materials and methods

### 2.1 Patient-specific image biomarkers from MRI

#### 2.1.1 Patient-specific data

A retrospective study of PCa patients was conducted at the Hospital Universitario y Politécnico de La Fe de Valencia (from now on HULAFE) between 2015 and 2020, within the ProCanAid research project (PLEC 2021-007709). To be included in the study, the following criteria should be met: patients should be men over 18 years of age with a confirmed diagnosis through a positive biopsy, should have received treatment at the specified hospital, should have undergone at least two mpMRI, and should possess the necessary clinical information (GS, biopsy data, etc.). In this study, we focus on modeling tumour growth in patients under AS. A total of six patients (N = 6) meet these criteria and are included in the study. Detailed imaging acquisition information for each patient is provided in the Supplementary Material.

All patients included in this study have been diagnosed with PCa via biopsy or had a biopsy performed close to the time of their initial mpMRI scan. Each patient has a GS of (3 + 3) 6. Information on the tumour burden was obtained from the biopsy samples, providing the average percentage of tumour cells present in each biopsy cylinder for each patient. In addition, these patients have been clinically monitored with PSA measurements to track disease progression.

#### 2.1.2 mpMRI data pre-processing

**FIGURE 1:**
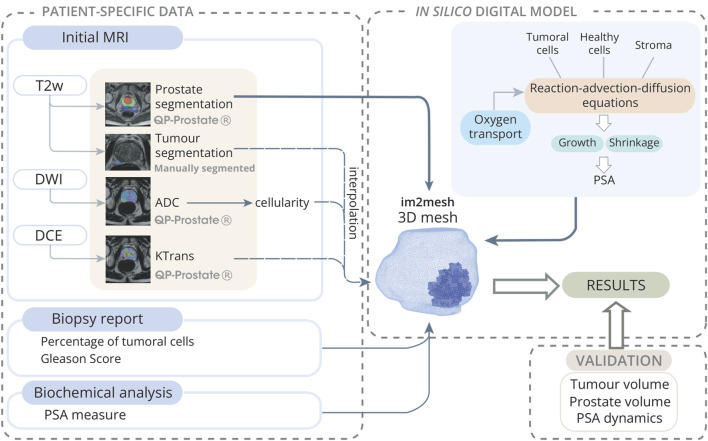
Model initialization scheme towards the digital twin: Patient-specific data is collected from an mpMRI study, along with biopsy and biochemical reports. MRIs are analyzed using QP-Prostate®software to obtain prostate segmentation from T2w-sequences, ADC maps from DWI sequences, and $K^{\mathrm{Trans}}$ maps from DCE sequences. The tumour is manually segmented by radiologists. The ADC provides an estimate of cellularity, while $K^{\mathrm{Trans}}$ offers insights into the vascularization of the prostate. The Python library im2mesh (Sainz-DeMena et al., 2022) is employed to reconstruct the 3D geometry of the prostate and generate the volumetric FE mesh. Subsequently, cellularity and $K^{\mathrm{Trans}}$ values are interpolated to the integration point of each element in the mesh to obtain the initial parameters of the model. Additionally, a mask is derived from nosological segmentations to identify mesh elements containing tumour cells. The Gleason Score, obtained from biopsies, provides information on the degree of cell differentiation and the overall percentage of tumour cells in each cylinder. With this data, the model is initialized for computational simulations, which are subsequently validated with clinical data.The model considerate multiple constituents (healthy, tumoural cells and stroma) and the transport of oxygen, thus the growth or shrinkage is given by proliferation or death of healthy and tumoral cells and production or remodellation of stroma. All this processes are defined by reaction-advection-diffusion equations.

Image pre-processing is performed using QP-Prostate®(Quibim S.L.) software. This software’s processing chain includes spatial smoothing, motion correction, and intra-series registration of the dynamic volumes of DWI and DCE, followed by inter-series registration of DWI and DCE to the T2W sequence. From the smoothed and motion-corrected DWI, ADC maps are calculated for each slice of the sequence. Similarly, the smoothed and motion-corrected DCE undergoes pharmacokinetic analysis (Jimenez-Pastor et al., 2023) (Figure 1). Specifically, the Standard Tofts Model (STM) is applied to the DCE and fitted using the least squares method (LSM). Among the parameters obtained, we focus on $K^{\mathrm{Trans}}$ , which measures the combination of bloodflow, vessel permeability and vessel surface on each voxel, i.e., it provides useful insight into the vascularisation of the prostate (Sainz-DeMena et al., 2024).

The ADC is a measure of the magnitude of diffusion (of water molecules) within tissue. It have been shown that it inversely correlates with the tissue cellularity so a quantification of the cellularity present in the tissue can be obtained by means of the Equation 1 (Atuegwu et al., 2013):

$$cellularity=\frac{ADC_{w}-ADC\left(x\right)}{ADC_{w}-ADC_{\min}}$$ (1)

where $ADC_{w}$ is the ADC value of free water molecules [ $ADC_{w}=3\cdot 10^{-3}mm^{2}/s$ (Atuegwu et al., 2013)] and $ADC_{\min}$ is the minimun ADC value captured. In this study, to ensure consistency of cellularity values between different patients and different follow-up MRIs, the $ADC_{\min}$ is standardized to 0.

#### 2.1.3 Prostate and tumour segmentations

The prostate gland is automatically segmented in the T2w sequence with QP-Prostate®(Quibim S.L.) software. This software utilizes an artificial intelligence (AI) algorithm founded on Convolutional Neural Networks (CNNs).

Conversely, radiologists at HULAFE perform manual segmentation of the tumoral lesions, ascertaining the quantity of lesions discernible in the T2w sequence. In the patients selected for the study, only one lesion was found for each T2w image.

#### 2.1.4 Mesh generation and interpolation of the data

The Python library im2mesh (Sainz-DeMena et al., 2022) is used to reconstruct the 3D geometry of the different prostates and to generate the volumetric FE mesh needed for the later simulations. Im2mesh library takes prostate and tumour segmentations, makes an estimation of intermediate and missing slices, reconstruct the surface and generate a volumetric mesh. Then, the cellularity and the $K^{\mathrm{Trans}}$ are automatically interpolated into the integration point of each element of the FE mesh to obtain the initial parameters of the model. Through lesion segmentation, a binary mask is created, corresponding to the number of elements within the mesh. Each element is assigned a value of 1 if located within the tumour region, and 0 otherwise. This procedure is termed interpolation, as it entails the transference of data from the lesion segmentation onto the finite element mesh representing the prostate (Figure 1).

#### 2.1.5 Integration of the data into the model

To integrate the image biomarkers into the model presented in the Section 2.2, additional calculations are needed. The ADC values provide a quantification of the cellularity in each element. In elements where both healthy cells and tumour cells coexist (i.e., where the tumour mask is 1), it is essential to distinguish the percentage of cellularity between these 2 cell types. This distinction is made using biopsy data on tumour load, which indicates the percentage of cellularity corresponding to tumour cells $\left(p_{t}\right)$ . For stroma density, it is assumed that the fraction not occupied by cells is filled by stroma. This cellularity is physiologically unfeasible to reach a value of 1, as such a measure would imply the complete absence of stroma, which is not possible for the cells to survive. Subsequently, to calculate cell densities, it is assumed that the carrying capacity $\left(\rho_{c}^{i}\right)$ represents the maximal cell population within a fully saturated element (Equation 2). This capacity varies between healthy and tumoural tissues, with the latter assumed to possess a greater capacity due to the cells’ propensity to deform and fill luminal spaces. The carrying capacity was determined following the methodology outlined by Atuegwu et al. (2013), assuming the number of cells that would fit in an element of 1 $mm^{3}$ volume (Table 1).

$$\begin{array}{c} \rho^{t}=cellularity\cdot p_{t}\cdot \rho_{c}^{t}\\ \rho^{h}=cellularity\cdot \left(1-p_{t}\right)\cdot \rho_{c}^{h}\\ \rho^{s}=\left(1-cellularity\right)\cdot \rho_{c}^{s} \end{array}$$ (2)

where $\rho^{t}$ , $\rho^{h}$ and $\rho^{s}$ are the population densities for tumoral cells, healthy cells and stroma.

**TABLE 1:** Parameters of the model.

Symbol	Parameter	Value	Units	References
$k_{g}$	Proliferation rate	4.03 $\cdot 10^{-3}$	$day^{-1}$	Optimised
k	Carrying capacity corrected	0.57	-	Optimised
$\rho_{c}^{t}$	Tissue tumour cell carrying capacity	100 $\cdot 10^{3}$	$cells\cdot mm^{-3}$	Atuegwu et al. (2013)
$\rho_{c}^{h}$	Tissue healthy cell carrying capacity	75 $\cdot 10^{3}$	$cells\cdot mm^{-3}$	Atuegwu et al. (2013)
$\rho_{c}^{s}$	Tissue stroma carrying capacity	1.50	$g\cdot mm^{-3}$	Agosti et al. (2018)
$k_{d}$	Death rate	0.02	$day^{-1}$	Lorenzo et al. (2016)
$\theta_{p}^{t}$	Proliferation oxygen threshold tumoral cells	3500	pmol	McKeown (2014)
$\theta_{d}^{t}$	Necrosis oxygen threshold tumoral cells	3000	pmol	McKeown (2014); Sosa-Marrero et al. (2021)
$\theta_{p}^{h}$	Proliferation oxygen threshold healthy cells	4000	pmol	McKeown (2014)
$\theta_{d}^{h}$	Necrosis oxygen threshold healthy cells	3300	pmol	McKeown (2014); Sosa-Marrero et al. (2021)
$\theta_{p}^{s}$	Oxygen threshold of stroma production	4000	pmol	McKeown (2014)
$\theta_{d}^{s}$	Necrosis oxygen threshold stroma	3900	pmol	McKeown (2014); Sosa-Marrero et al. (2021)
$A_{o}^{t}$	Maximum oxygen consumption rate	25.50	$pmol\cdot s^{-1}$	Mpekris et al. (2015)
$k_{o}^{t}$	Oxygen concentration at one-half of the total consumption term	4.64	$pmol\cdot s^{-1}$	Mpekris et al. (2015)
$A_{o}^{h}$	Oxygen uptake of healthy cells	25.50	$pmol\cdot s^{-1}$	Mpekris et al. (2015)
$k_{o}^{h}$	Oxygen uptake of healthy cells	4.64	$pmol\cdot s^{-1}$	Mpekris et al. (2015)
$\rho_{b}^{o}$	Blood oxygen concentration	4124	pmol	Mpekris et al. (2015)
$K_{\mathrm{stiffness}}$	Stiffness of prostate surroundings	14	kPa	Optimised
$E^{t}$	Young’s modulus of tumoral cells	3	kPa	Faria et al. (2008); Lekka et al. (2012)
$E^{h}$	Young’s modulus of healthy cells	5	kPa	Faria et al. (2008); Lekka et al. (2012)
$E^{s}$	Young’s modulus of stroma	30	kPa	Bharatha et al. (2001)
ν	Poisson’s ratio	0.40	-	Lorenzo et al. (2019a)
$k_{v1}$	Maximum growth rate	0.95	-	Optimised
$k_{v2}$	Incremental growth factor	0.1	-	Optimised
$k_{d1}$	Shrinking rate	3	-	Hervas-Raluy et al. (2024)
$\alpha^{t}$	PSA production rate of tumoral cells	0.96	$ng\cdot mL^{-1}\cdot day^{-1}$	Optimised
$\alpha^{h}$	PSA production rate of healthy cells	8.02 $\cdot 10^{-3}$	$ng\cdot mL^{-1}\cdot day^{-1}$	Optimised
γ	Tissue PSA decay rate	2.17 $\cdot 10^{-4}$	$day^{-1}$	Optimised
$\gamma_{s}$	Blood PSA decay rate	1.44 $\cdot 10^{-4}$	$day^{-1}$	Optimised

### 2.2 Mathematical model

A multispecies PDE reaction-advection-diffusion model coupled with continuous media mechanics is presented for the simulation of PCa growth, encompassing both tumour growth dynamics and its impact on the entire prostate gland. This multispecies model aims to represent the phenomenological behaviour of PCa and its cellular processes, including proliferation, differentiation and apoptosis. Based on these processes, the model simulates the growth of the prostate and tumour geometry and the dynamics of PSA.

#### 2.2.1 Constituents

The prostate consists mainly of glandular epithelial cells (luminal and basal cells) and stroma, which are organised into lumens and ducts for the secretion of prostatic fluid. On the one hand, when these cells undergo mutations, they lose their healthy properties and their proliferative capacity increases, thus disrupting the formation of the glandular structure (Henry et al., 2018; Rebello et al., 2021). On the other hand, the stroma plays an essential role in the interaction between cells and the microenvironment, and its mechanical properties have been shown to play an important role in tumour growth and response (Niu and Xia, 2009). The environmental and mechanical conditions surrounding cells are essential in the control of cell populations. An example of such environmental factors would be oxygen and nutrients, normally supplied by blood vessels via diffusion (Vaupel and Kelleher, 2013; Mpekris et al., 2015; Kay, 1983). In this model, for simplicity, oxygen is considered as the only factor. This model considers mainly three constituents: healthy cells, tumoral cells and stroma; thus, each of these constituents is defined by its density $\left(\rho^{i}\right)$ . The dynamics governing the evolution of population densities are governed by the principles of mass conservation, given by advection-reaction-diffusion (Equation 3):

$$\begin{array}{ll} \frac{\partial \rho^{i}}{\partial t}-▽\cdot \left(D▽\rho^{i}\right)+▽\left(\rho^{i}\frac{\partial u}{\partial t}\right) & =\rho^{i}k_{g}\frac{1}{1+\beta^{i}}\left(\frac{k\rho_{c}^{i}-\rho^{i}}{k\rho_{c}^{i}}\right)H\left(\rho^{o}-\theta_{p}^{i}\right)\\ & \;-\rho^{i}k_{d}H\left(\theta_{d}^{i}-\rho^{o}\right) \end{array}$$ (3)

where i are the three constituents (*t* = tumoral cells; *h* = healthy cells; and *s* = stroma). The terms in the left side of the equation correspond to the temporal rate of change of the *i*-th population density, the diffusive term and the convective term and the ones in the right side correspond to the proliferation and the death term, respectively. The proliferation term depends on $k_{g}$ , which is the proliferation rate, $\rho_{c}^{i}$ , which is the tissue carrying capacity, k , which is the corrected carrying capacity, that acts as a constraint to prevent cells unlimited cell proliferation, and $\beta^{i}$ , which is the cell dependent parameter being 1 for healthy cells and 0 for tumoral cells. $k_{g}$ is uniformly applied across all constituents, based on the premise that $\beta^{i}$ plays a crucial role in modulating growth by diminishing the proliferation rate within healthy tissue. The $k_{d}$ constant defines the process of death due to hypoxia. This parameter can be defined according to the aggressiveness of the cancer (Lorenzo et al., 2016). As this work is focused on low to intermediate risk patients, the chosen parameter is selected in Table 1. Both cell proliferation and cell death processes are given by the Heaviside function H , depending on the oxygen concentration $\rho^{o}$ , where $\theta_{p}^{i}$ and $\theta_{d}^{i}$ represent the oxygen concentration threshold of proliferation and death, respectively, of each constituent i . Spatial saturation is also limited by the k parameter. This parameter ensures that the concentration of the constituents never exceeds 1. It also preserves necessary space for stroma, preventing cellular overpopulation, which is vital for cell survival. The species displacement, u , results from both tumour growth and its deformation as it interacts with the surrounding tissues and organs at position **x** and time t. As our model focuses on predicting the tumour growth in a confined manner, the diffusive term (D) , i.e., cell migration, is underestimated in these equations for simplicity, as both healthy and tumoral cell migration is assumed to be low compared to other cell processes (Pienta and Loberg, 2005).

#### 2.2.2 Oxygen transport

For the sake of simplicity, only oxygen and PSA (see Section 2.2.3) transport has been considered. The model can be expanded to integrate additional factors such as glucose or other nutrients. However, due to the complexity of modeling all mechanisms involved in tumour growth, oxygen has been selected as a representative factor for all these nutrients. Oxygen concentration is mainly determined by its exchange with blood, which depends specifically on prostate vascularisation represented by $K^{\mathrm{Trans}}$ , and a negative term accounting for cell consumption. Transport equation is defined as (Equation 4).

$$\frac{\partial \rho^{o}}{\partial t}-▽\cdot \left(D^{o}▽\rho^{o}\right)=K^{\mathrm{Trans}}\left(\rho_{b}^{o}-\rho^{o}\right)-\overset{h,t}{\underset{i}{\sum }}\frac{A_{o}^{i}\rho^{o}}{k_{o}^{i}+\rho^{o}}\frac{\rho^{i}}{\rho_{c}^{i}}$$ (4)

where $K^{\mathrm{Trans}}$ is the oxygen extravasation parameter, which defines the supply rate; and $\rho_{b}^{o}$ is the oxygen concentration in blood. The coefficients $A_{o}^{i}$ and $k_{o}^{i}$ (where i=h,t ) represent the oxygen consumption of the different constituents of the model. More specifcally, $A_{o}^{i}$ is the maximum species consumption rate and $k_{o}^{i}$ is the species concentration at which the total consumption term is one-half of the total consumption term. These parameters are considered equal for both healthy and tumoral cells (Table 1), as a first simplified approach that aids in the development and analysis of the model. Although there are metabolic differences between these cell types, a single parameter is a reasonable approximation for studies primarily focused on tumour growth dynamics. Since the model’s predictions are not significantly affected by variations in this parameter, this simplification is beneficial in reducing complexity and computational cost without greatly impacting the model’s accuracy. While it is widely recognized that diffusion influences the distribution of species such as oxygen within a tumour (Greenspan, 1972; Wang et al., 2015), in well-vascularised tissue active transport through blood vessels may limit diffusion (Hervas-Raluy et al., 2024). Furthermore, it is crucial to acknowledge the considerable computational resources required to compute the diffusion process. According to our calculations, the associated costs are estimated to be around twenty times higher in cost.

#### 2.2.3 PSA simulation

The normal prostate architecture keeps PSA tightly confined, so that only a small proportion leaks into the circulatory system and a major part is delivered to the urethra (Sävblom et al., 2005). However, in the presence of cancer, this structure disrupts, as abnormal proliferation of tumoral cells occurs and obstructs the lumens and ducts through which PSA is secreted. Consequently, the PSA produced cannot reach the urethra, accumulating in the tissue, and leading to increased leakage into the bloodstream (Lilja et al., 2008). As a first approach, the model considers the elevation of PSA in the tissue as a consequence of the lumen disruption and leakage by tumoral cells. Therefore, the increase of PSA in the tissue is mainly proportional to the concentration of tumoral and healthy cells with different effects. The intravasation from PSA tissue to blood depends on the prostate vascularization. Thus, $K^{\mathrm{Trans}}$ is considered the principal physical property driving this exchange, depending on the difference in concentration between PSA in the tissue and in blood. Equation 5 and Equation 6 describe the dynamic of PSA tissue (P) and PSA serum in blood $\left(P_{s}\right)$ , respectively.

$$\frac{\partial P}{\partial t}=\alpha^{h}\frac{\rho^{h}}{\rho_{c}^{h}}+\alpha^{t}\frac{\rho^{t}}{\rho_{c}^{t}}-K^{\mathrm{Trans}}\left(P-P_{s}\right)-\gamma P$$ (5)

$$\frac{dP_{s}}{dt}=\int {\tilde{K}}^{\mathrm{Trans}}\left(P-P_{s}\right)dv-\gamma_{s}P_{s}$$ (6)

where $\alpha^{t}$ and $\alpha^{h}$ are the production ratio of PSA by tumoral and healthy cells respectively, being $\alpha^{t}$ larger than $\alpha^{h}$ . γ and $\gamma_{s}$ are the natural decay parameters of PSA in the tissue and in blood respectively. Serum PSA in blood is the sum of the exchange between tissue and blood in each infinitesimal part of the prostate (dv) , and ${\tilde{K}}^{\mathrm{Trans}}$ is $K^{\mathrm{Trans}}$ per unit volume $\left({\tilde{K}}^{\mathrm{Trans}}=\frac{K^{\mathrm{Trans}}}{dv}\right)$ .

### 2.3 Kinematics of growth

Let Ω be the prostate in a three-dimensional space, so that a movement $\varphi :\Omega_{0}\to \Omega_{t}$ maps a material or reference configuration $\Omega_{0}$ point to a current configuration $\Omega_{t}$ by means of Equation 7.

$$x\left(X,t\right)=\varphi \left(X,t\right)$$ (7)

The model assumes that a mechanical body consist of i−th constituents that are characterised by their density and share each differential volume element. This implies that these constituents deform together, presenting the same deformation gradient tensor F . The deformation gradient tensor in the theory of continuous non-linear mechanics maps a material point X from the reference configuration $\left(\Omega_{0}\right)$ to an spatial point x in the current configuration $\left(\Omega_{t}\right)$ at any given time t (Equation 8).

$$F=\frac{\partial x}{\partial X}$$ (8)

**FIGURE 2:**
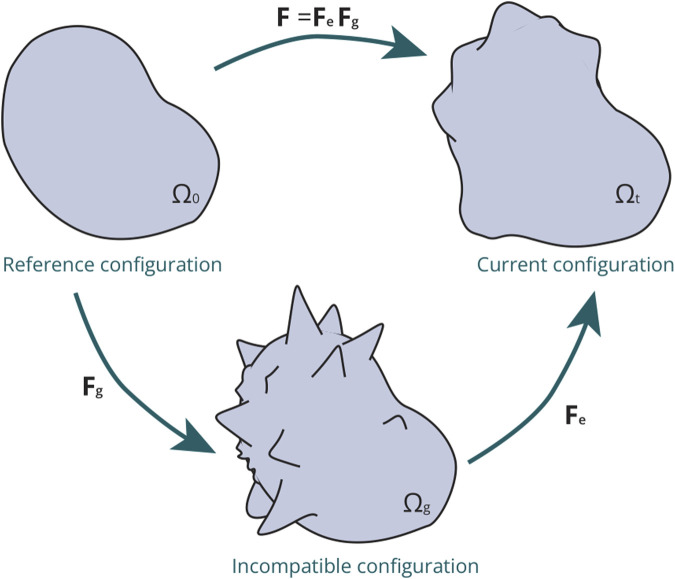
Schematic representation of the multiplicative decomposition applied to the deformation gradient **F** within a continuous framework to characterise growth: The transition from the reference configuration $\Omega_{0}$ to the current configuration $\Omega_{t}$ involves the initial calculation of an intermediate configuration $\left(\Omega_{g}\right)$ through $F_{g}$ associated with volumetric growth. This intermediate configuration is not a physically experienced state by the tissue; rather, it represents an incompatible configuration. Subsequently, the current configuration is attained through the elastic component $F_{e}$ , which characterizes the mechanical response of the tissue.

Additionally, the multiplicative decomposition of the deformation tensor is used to describe the kinematics of growth (Equation 9).

$$F=F_{e}^{i}\cdot F_{g}^{i}$$ (9)

where the total deformation gradient tensor F is accounted for by an elastic deformation gradient tensor $\left(F_{e}^{i}\right)$ , related to the stress response of the material, and an inelastic deformation gradient tensor $\left(F_{g}^{i}\right)$ , connected to the volumetric growth. Therefore, to attain the current configuration $\Omega_{t}$ from the reference configuration $\Omega_{0}$ , an intermediate configuration related to volumetric growth is first calculated $\left(\Omega_{g}\right)$ . This intermediate configuration is not a physical state actually experienced by the tissue, it is usually an incompatible configuration (Comellas et al., 2018). The current configuration is then reached through the elastic deformation gradient component $F_{e}$ , which defines the mechanical response of the tissue (Figure 2).

The growth component $F_{g}^{i}$ is set to be homogeneous and isotropic so that Equation 10.

$$F_{g}^{i}=\lambda_{g}^{i}I$$ (10)

where $\lambda_{g}^{i}$ is the growth stretch ratio of every *ith* constituent. This factor governs inelastic deformation, which is assumed to be due to changes in mass production over the tissue corrected carrying capacity $\left(\rho_{c}^{i}\right)$ , in such a way that the growth takes place if $\rho_{g}^{i}>\rho_{0}^{i}$ and, on the contrary, resorption if $\rho_{g}^{i}<\rho_{0}^{i}$ [Equation 11 (Hervas-Raluy et al., 2024)], being $\rho_{0}^{i}$ and $\rho_{g}^{i}$ the constituent densities in the reference and incompatible configuration, respectively.

$$\lambda_{g}^{i}=\left\{\begin{array}{c} 1+k_{d1}\left(\frac{\rho_{g}^{i}-\rho^{i}}{\rho^{i}}\right)\;if\;\rho_{g}^{i}\leq \rho_{0}^{i}\\ k_{v1}+\frac{k_{v2}}{1+exp\left(-\frac{\rho_{g}^{i}}{k\rho_{c}^{i}}\right)}\;if\;\rho_{g}^{i}>\rho_{0}^{i} \end{array}\right.$$ (11)

where $k_{v1}$ and $k_{v2}$ are constants defining the growth rate and $k_{d1}$ governs the change of volume due to mass resorption.

The consideration of mass change is essential for accurately establishing stress-strain relations to replicate tumour growth. As the mass need to be conserved from $\Omega_{g}$ to $\Omega_{t}$ , the formulations for the densities of the grown mass $\left(m^{i}\right)$ concerning different configurations are expressed in Equation 12 and Equation 13.

$$dm^{i}=\rho_{g}^{i}dv_{g}^{i}$$ (12)

$$dm^{i}=\rho^{i}dv$$ (13)

where $v_{g}^{i}$ is the volume in $\Omega_{g}$ and v the volume in $\Omega_{t}$ . Therefore, $\rho_{g}^{i}$ can be rewritten as in Equation 14, where $J_{e}^{i}$ is the volume ratio given by Equation 15.

$$\rho_{g}^{i}=\rho^{i}J_{e}^{i}$$ (14)

$$J_{e}^{i}=detF_{e}^{i}=\frac{dv}{dv_{g}^{i}}$$ (15)

All the constituents are assumed to be linear elastic and the properties of each element (j) are estimated by the rule of mixtures (Equation 16).

$$E_{j}=\sum E^{i}\frac{\rho^{i}}{\rho_{c}^{i}}$$ (16)

### 2.4 Implementation

#### 2.4.1 Numerical strategy

**FIGURE 3:**
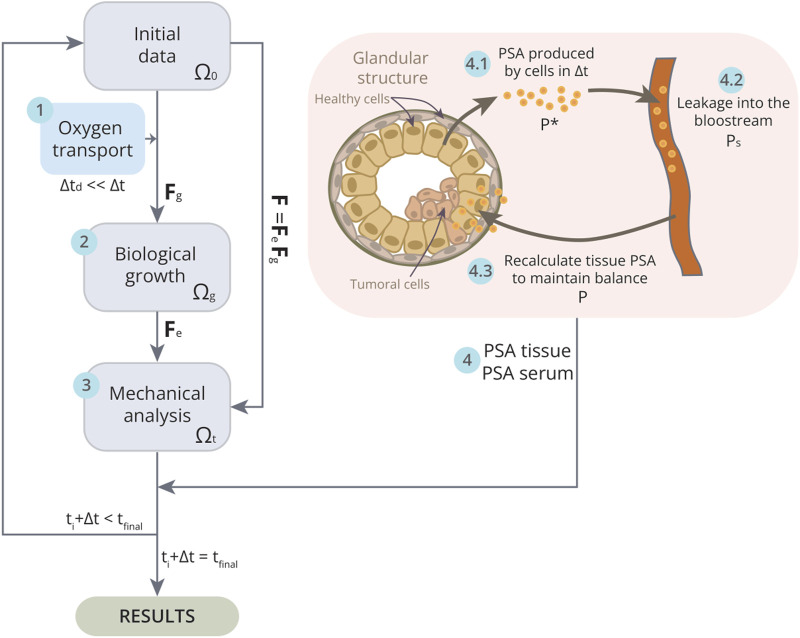
Schematic of the implementation of the model in one iteration Δt . First, the resulting oxygen concentration in each element at the beginning of the iteration is calculated with the transport equation for a $\Delta t_{d}$ (1). Since $\Delta t_{d}$ is much smaller than Δt , it is assumed that a stationary state is reached that lasts until the end of the iteration. Subsequently, the biological growth of each of the i constituents in Δt is updated taking into account the previously calculated oxygen concentration, reaching the intermediate incompatible configuration $\left(\Omega_{g}\right)$ (2). To solve the mechanical problem (3), the software Ansys®Academic Research Mechanical, Release 19.2 is used, thus reaching the current configuration $\left(\Omega_{t}\right)$ . Finally, once the population densities in Δt are known, the PSA in tissue and blood are estimated (4). The cells in the figure represent the typical structure formed by glandular cells in the prostate, which are arranged to form lumens that secrete prostatic fluid to fulfill their glandular function. However, tumour cells proliferate, occupying and obstructing these lumens (darker cells in the figure), thereby preventing the secretion of PSA into the seminal ducts and causing a larger portion of PSA to leak into the blood. To calculate PSA dynamics, the total PSA production or decay $\left(P^{*}\right)$ during the time increment Δt (4.1) is first estimated. Then, the serum PSA concentration in the blood $\left(P_{s}\right)$ is calculated at the stationary state (4.2). Eventually, the required tissue PSA level for equilibrium with the specified serum PSA level is recalculated (P) (4.3). When the iteration has been completed, it is checked if the final time $\left(t_{\mathrm{final}}\right)$ of the simulation has been reached, in which case the results will be obtained. If not, a new iteration is restarted, taking as initial data the final data of the previous iteration.

The proposed model is solved using the FE method. The mechanical analysis is evaluated decoupled from the biological one assuming growth incompatibility. Therefore, the growth tensor is programmed in Python 3.7.12, while the elastic contribution is computed in the commercial FE software Ansys®Academic Research Mechanical, Release 19.2, implemented by means of an ANSYS APDL (Figure 3). The thermoelastic expansion equations are used as an analogy governing volumetric changes in expansion and contraction processes to simulate tumour or prostate growth or shrinkage (Vujošević and Lubarda, 2002), so the growth or degrowth would be indicated in the ANSYS APDL file as a increment of temperature positive or negative, respectively ( $J_{g}^{i}={\left(1+\alpha \Delta T\right)}^{3}$ , being α a coefficient expansion ratio of 1 and ΔT the increment of temperature) (Hervas-Raluy et al., 2024). By neglecting diffusion and decoupling the convective term, Equation 3 can be simplified by transforming the PDE into an ordinary differential equation (ODE), which is solved by the explicit Euler method programmed in Python. On the other hand, the Newton-Raphson method with an implicit iterative method is used to solve the growth modulus by means of Ansys®.

It is assumed that each volume element contains a mixture of two structurally significant components, namely, the cells and the stroma, so that the interaction between cells and stroma is modelled. The cell populations are categorized into healthy and tumour cells, coexisting within the tumour, while the remainder of the prostate consists solely of healthy cells. Therefore, the model operates on the assumption that cellular and stromal-based matrix support the same mechanical strains, therefore as they have different mechanical properties they present different stresses. Consequently, the total stress supported by the prostate is considered the sum of both cellular and stromal contributions. To represent this, the prostate volume is discretised into two overlapping meshes with the same number of elements and sharing nodes. The mesh is composed of three-dimensional linear tetrahedral linear elements generated using im2mesh Pyhton library (Sainz-DeMena et al., 2022). The average size element is 2.5 mm for all the patients and a time step of 10 days is set.

The prostate is assumed to be located in the space bounded by the surrounding organs, mainly rectum and bladder. These organs undergo daily fluctuations due to their normal functioning, which alter their physical characteristics and make it challenging to select a material stiffness that precisely mimics the surrounding anatomical conditions. However, for this purpose, the material behaviour of these organs can be simplified to follow elastic behaviour (Dall et al., 1993; Chai et al., 2011). Therefore, to replicate the stiffness of these surrounding tissues, springs $\left(K_{\mathrm{stiffness}}\right)$ are set in the direction normal to the prostate as contour conditions.

#### 2.4.2 Multiscale temporal implementation

Cellular processes such as proliferation or death occur over a broader time span than other phenomena such as the transport of substances like oxygen and the leakage of PSA into the blood. Therefore, in order to fully integrate all these phenomena and the interaction between the cells and these substances, a multiscale temporal algorithm has been modelled. For both oxygen transport and PSA leakage, it is assumed that the equilibrium state of these substances is reached before the time increment (Δt) given for each iteration of the simulation.

First, the arrival of oxygen is simulated until an equilibrium state is reached (Figure 3, step 1). By removing the diffusion term from Equation 4, an immediate equilibrium between concentrations would theoretically be attained. Nonetheless, a small time interval, denoted as $\Delta t_{d}$ , is required for this exchange to transpire in practice. Once equilibrium is established, cellular processes are calculated and population densities are updated. The resolution of this equation employs the 8th-order explicit Runge-Kutta numerical method for ODEs. The initialisation of the oxygen concentration begins with the application of the transport equation (Equation 4), which assumes that the initial oxygen concentration in the prostate is zero. This premise facilitates the modelling of oxygen transfer from the vasculature to the tissue at the beginning of the first step.

To address the multiscale temporal problem associated with PSA dynamics, it is also assumed that the equilibrium between PSA tissue concentration and PSA serum in blood is achieved well before the completion of the time increment Δt , resulting in a stationary state (Figure 3). To achieve this, the total amount of PSA produced or decayed $\left(P^{*}\right)$ in that time increment Δt is first estimated (Equation 17). Subsequently, the PSA serum concentration in the blood at the point of stationary state is calculated based on the assumed $P^{*}$ (Equation 18). Finally, the PSA level required in the tissue to attain the specified serum PSA level in the blood and maintain equilibrium is recalculated (Equation 19). In this way, the computation can be simplified by using the explicit Euler method for ODEs.

$$\frac{\Delta P^{*}}{\Delta t}=\alpha^{h}\frac{\rho^{h}}{\rho_{c}^{h}}+\alpha^{t}\frac{\rho^{t}}{\rho_{c}^{t}}-\gamma P$$ (17)

$$\frac{\Delta P_{s}}{\Delta t}=\sum K^{\mathrm{Trans}}\left(P^{*}-P_{s}\right)-\gamma_{s}P_{s}=0$$ (18)

$$\frac{\Delta P}{\Delta t}=\alpha^{h}\frac{\rho^{h}}{\rho_{c}^{h}}+\alpha^{t}\frac{\rho^{t}}{\rho_{c}^{t}}-K^{\mathrm{Trans}}\left(P-P_{s}\right)-\gamma P=0$$ (19)

Although the implementation assumes an equilibrium between blood and tissue, this does not imply that the values of P and $P_{s}$ are equal at equilibrium, as there are multiple factors that influence this equilibrium. These equations are proposed on the basis of heterogeneity in $K^{\mathrm{Trans}}$ , which reflects the premise that the exchange between tissue and blood is heterogeneous and vascular-dependent. The $K^{\mathrm{Trans}}$ parameter summarises this idea, indicating that elevated $K^{\mathrm{Trans}}$ values correspond to an enhanced exchange capacity of substances within a specific region. The initial tissue PSA is computed using Equation 19 which recalculates the tissue PSA from the serum PSA concentration. PSA serum values, derived from biochemical analyses for all patients, are adjusted via an exponential curve. The initial tissue PSA is determined by the PSA serum value on the fitted exponential curve corresponding to the date of the first MRI. This approach guarantees consistency and accurate reflection of the serum PSA measurements in the initial tissue PSA values.

### 2.5 Acquisition of model parameters

The parameters used in the model are summarised in Table 1. Most of these parameters are taken from the literature. Nevertheless, certain parameters, like the proliferation rate $\left(k_{g}\right)$ , the corrected carrying capacity (k) , the incremental growth factor $\left(k_{v2}\right)$ , the stiffness of the surrounding tissue $\left(K_{\mathrm{stiffness}}\right)$ or those related with PSA dynamics, are particularly specific to the model or display considerable variability across existing research, making difficult to assign definitive values. To address this, an optimization process is performed by means of the Python Optuna library (Akiba et al., 2019). Optuna is a widely-used hyperparameter optimization framework that leverages advanced techniques to optimize machine learning models. It primarily employs the Tree-structured Parzen Estimator (TPE) as its default sampler, utilizing a Bayesian optimization algorithm (Akiba et al., 2019). A multi-objective optimization process is chosen as it allows for adjusting the best parameters for several patients simultaneously, balancing the objective functions. Following examples from Optuna’s documentation, the optimization algorithm is developed in Python. This algorithm runs the tumour growth model a specified number of times, referring to each iteration as a trial. In each trial, the parameters to be adjusted are varied within a defined space, and the model is run for each patient with that set of parameters. Therefore, the final objective is to find the set of parameters that minimize simultaneously an objective function for each patient. Furthermore, Optuna inherently performs a sensitivity analysis throughout the optimization process, utilizing the Functional ANOVA evaluator (Akiba et al., 2019). As it adjusts parameters across trials, Optuna evaluates how variations in each parameter influence the objective function, providing insights into parameter importance.

Two optimization processes are performed to comprehensively refine the model. Initially, parameters governing PCa growth undergo optimization with the objective of minimizing the disparities between the MRI data and computational outcomes in prostate volume, tumour volume, mean prostate cellularity, and mean tumour cellularity. Following the acquisition of optimal values, a secondary optimization is conducted to determine parameters that most accurately align with PSA dynamics.

#### 2.5.1 PCa growth model parameters optimization

The parameters to optimise are the proliferation rate $\left(k_{g}\right)$ , the corrected carrying capacity (k) , the incremental growth factor $\left(k_{v2}\right)$ and the stiffness of the surrounding tissue $\left(K_{\mathrm{stiffness}}\right)$ . Table 2 shows the ranges within which these parameters are optimized. $k_{g}$ range is derived from a study in male Sprague Dawley rats where the proliferation rate of tumour cells was calculated as a function of the concentration of testosterone-activated androgen receptor (AR) (Jain et al., 2011). The k range is determined by considering the upper limit of cellularity permissible within a biological context for a given element. This ensures that the element’s cellularity remains within a physiologically realistic range. The estimation of $k_{v2}$ is conducted by defining the growth threshold of an element, which is directly influenced by the density of its constituent components. This approach ensures that the element’s expansion is quantitatively aligned with its internal density parameters. Furthermore, the bladder and rectum, which are adjacent to the prostate, undergo daily fluctuations in their states. This alters their physical characteristics, posing a challenge in selecting a material stiffness that precisely mimics the surrounding anatomical conditions. The ranges given are obtained from (Dall et al., 1993; Chai et al., 2011).

**TABLE 2:** Combined parameters for optimization.

Symbol	Parameter	Range	Units	References
$k_{g}$	Proliferation rate	$\left[1\cdot 10^{-3}-0.1\right]$	$day^{-1}$	Jain et al. (2011)
k	Carrying capacity corrected	[0.5−0.9]	-	Estimated
$K_{\mathrm{stiffness}}$	Stiffness of prostate surroundings	[10−20]	kPa	Boubaker et al. (2009)
$k_{v2}$	Incremental growth factor	[0.05−0.1]	-	Estimated
$\alpha^{t}$	PSA production rate of tumoral cells	$\left[1\cdot 10^{-2}-1\right]$	$ng\cdot mL^{-1}\cdot day^{-1}$	Estimated
$\alpha^{h}$	PSA production rate of healthy cells	$\left[0-9\cdot 10^{-3}\right]$	$ng\cdot mL^{-1}\cdot day^{-1}$	Lorenzo et al. (2016); Jain et al. (2011)
γ	Tissue PSA decay rate	$\left[9\cdot 10^{-4}-9\cdot 10^{-2}\right]$	$day^{-1}$	Estimated
$\gamma_{s}$	Blood PSA decay rate	$\left[9\cdot 10^{-4}-9\cdot 10^{-2}\right]$	$day^{-1}$	Estimated

MRI patient data serve as the basis for model calibration, encompassing measurements such as prostate volume, tumour volume, mean prostate cellularity, and mean tumour cellularity from MRI follow-ups. The simulation results are then compared with these data, being the objective minimize any disparities between them. To facilitate this, the relative error is chosen as the objective function for minimization, as it accommodates errors measured in different units, calculated according to Equation 20.

$$E=\frac{1}{n}\overset{n}{\underset{i=1}{\sum }}\left|\frac{\left(\varepsilon_{o}^{i}-\varepsilon_{c}^{i}\right)}{\varepsilon_{o}^{i}}\right|$$ (20)

where i are the target values (prostate volume, tumour volume, mean prostate cellularity, and mean tumour cellularity for each follow up MRI), $\varepsilon_{o}^{i}$ is the objective value from clinical measurements and $\varepsilon_{c}^{i}$ is the computational result of each trial.

#### 2.5.2 PSA dynamics parameters optimization

After determining the optimal parameters for the PCa growth model, the subsequent stage involves optimizing the PSA dynamics. The parameters to optimise are the PSA production rate of tumoural cells $\left(\alpha^{t}\right)$ , PSA production rate of healthy cells $\left(\alpha^{h}\right)$ , the tissue PSA decay rate (γ) and the blood PSA decay rate $\left(\gamma_{s}\right)$ . Table 2 shows the ranges within which these parameters are optimized. A reduced range for PSA production rates is selected for healthy cells, predicated on the premise that in a healthy state, PSA is predominantly discharged into the prostate’s lumens and ducts, with minimal leakage into the surrounding tissue. Conversely, the proliferation of tumour cells leads to the occupation of lumen spaces, impeding the usual secretion pathways and resulting in increased PSA leakage into the tissue. Consequently, it is assumed that a small fraction of the PSA generated by healthy cells escapes into the tissue. The ranges for decay parameters γ and $\gamma_{s}$ were chosen through an iterative process to optimize the model’s fit with observed PSA dynamics.

Clinical measurements obtained through biochemical analysis of serum PSA in blood are used for optimizing the PSA dynamics parameters. Given the variability of these measurements due to various patient and environmental factors, the mean absolute error (MAE) is selected as the objective function for minimization, owing to its robustness against outliers (Equation 21).

$$MAE=\frac{1}{n}\overset{n}{\underset{i=1}{\sum }}\left|\left(\varepsilon_{o}^{i}-\varepsilon_{c}^{i}\right)\right|$$ (21)

where i are the different time points with PSA measurements, $\varepsilon_{o}^{i}$ is the objective clinical value and $\varepsilon_{c}^{i}$ is the computational result of each trial.

## 3 Results

### 3.1 Patient-specific data

This section introduces the patients who satisfy the study criteria detailed in Section 2.1.1.

**FIGURE 4:**
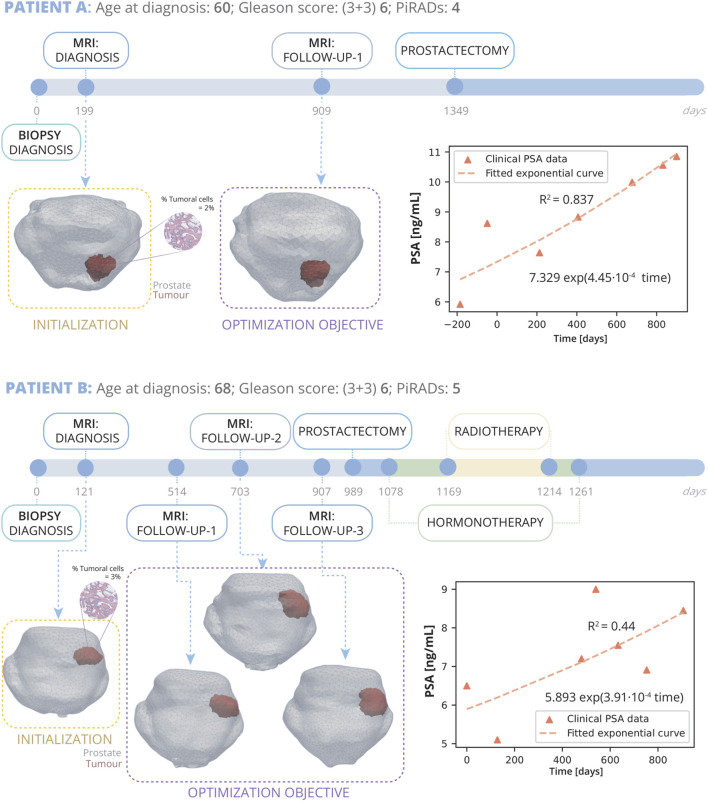
Patient A and B clinical history: Patient A, diagnosed at 60 with grade 4 PI-RADS, had a Gleason Score of 6 (3 + 3) and 2% tumour volume; underwent prostatectomy 1349 days post-diagnosis following a single follow-up MRI at day 909. Patient B, diagnosed at 68 with grade 5 PI-RADS, had a Gleason Score of 6 (3 + 3) and 3% tumour volume; received three MRIs on days 514, 703, and 907, and due to cancer’s progression, had a radical prostatectomy on day 989, followed by HT and RT. On the right, PSA measurements for both patients are displayed alongside their fitted exponential growth curves. Bellow the timeline, the FE mesh digital reconstructions of the prostate and tumours at diagnosis and follow-up MRIs is showed. The diagnosis MRI is utilized for the initialization of the model, while the subsequent follow up MRI serves for optimization.

Patient A (Figure 4) was diagnosed at the age of 60 years. He had no previous medical history of any other type of cancer and no close family members have had PCa. 199 days after biopsy diagnosis, he underwent an MRI scan which according to the PI-RADS report indicated a lesion in the medial posterior peripheral zone of the apex of the prostate with a grade 4. The biopsy analysis indicated a GS (3 + 3) 6 with an overall mean percentage of tumour volume of the extracted cylinders of 2%. He was not treated in the first instance and was included in AS with regular PSA blood tests. A follow up MRI was performed 710 days after diagnosis MRI, and finally, as clear growth was observed, he underwent radical prostatectomy surgery.

Patient B (Figure 4) was diagnosed at the age of 68 years. His clinical record also does not indicate the existence of any other cancer or that any family members have had PCa. Diagnosis MRI, taken 121 days after biopsy diagnosis, shows a grade 5 PI-RADS lesion in the medial posterior peripheral zone of the mid and base of the prostate. The diagnostic biopsy showed a GS of (3 + 3) 6 and an overall mean tumour volume percentage of the extracted cylinders of 3%. This patient was under active follow up with biochemical analysis of PSA in the blood for approximately 2.5 years until he underwent radical prostatectomy surgery. During this time, he underwent three follow up MRI scans. This patient also exhibited extraprostatic invasion and infiltration into the seminal vesicles, indicative of a more aggressive form of cancer. Therefore, in order to eliminate any possible remaining traces of cancer cells, subsequent to the prostatectomy, he also underwent HT and RT.

**FIGURE 5:**
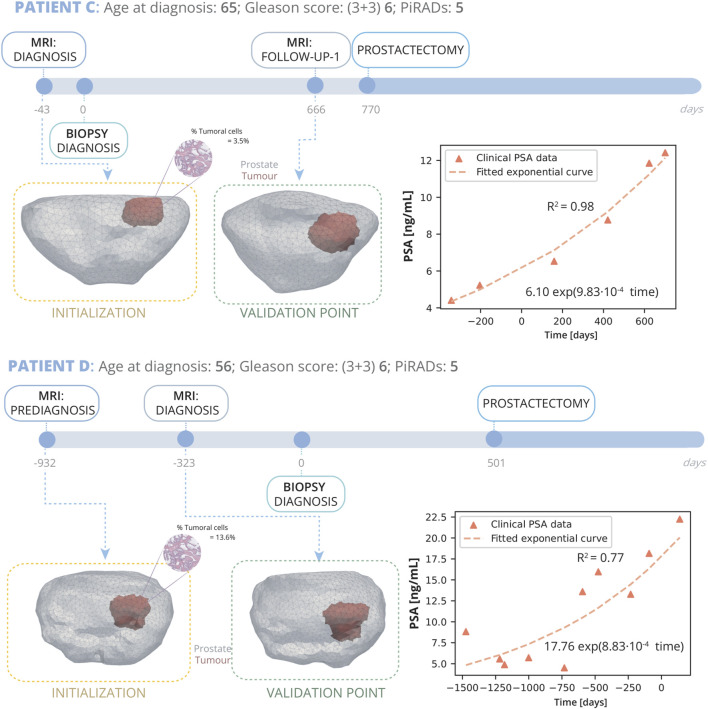
Patient C and D clinical history: Patient C, diagnosed at 65 with grade 5 PI-RADS, had a Gleason Score of 6 (3 + 3) and 3.5% tumour volume; underwent prostatectomy 770 days post-diagnosis following a single follow-up MRI at day 666. Patient D, diagnosed at 56 with grade 5 PI-RADS, had a Gleason Score of 6 (3 + 3) and 3% tumour volume; he underwent 2 MRI, one prediagnosis at day −932 and another for diagnosis at day −323, and due to cancer’s progression, had a radical prostatectomy on day 501. On the right, PSA measurements for both patients are displayed alongside their fitted exponential growth curves. Bellow the timeline, the FE mesh digital reconstructions of the prostate and tumours at diagnosis and follow-up MRIs is showed. The first MRI taken for each patient are used for the initialization of the model, while the subsequent MRI serves for validation.

Patient C (Figure 5) was diagnosed at the age of 65 years. Subsequent reviews of his medical history revealed no personal or family history of cancer. An MRI conducted 43 days prior to the biopsy pinpointed a PI-RADS grade 5 lesion located in both the mid and base sections of the peripheral zone, affecting posterior lateral and medial aspects. The biopsy returned a GS of 6 (3 + 3), with the tumorous tissue averaging 3.5% of the sampled cores’ volume. Initially opting not to undergo immediate treatment, Patient D was enrolled in AS, which included routine PSA monitoring. After a period of 666 days from the diagnosis biopsy, a subsequent follow-up MRI scan was performed showed significant tumour progression, leading to the decision for a radical prostatectomy at day 770.

Patient D (Figure 5) was diagnosed at the age of 56 years. Subsequent reviews of his medical history revealed no personal or familial history of cancer. An MRI conducted 932 days prior to the biopsy pinpointed a PI-RADS grade 5 lesion located in the mid medial section of the peripheral zone. The biopsy returned a GS of 6 (3 + 3), with the tumorous tissue averaging 13.6% of the sampled cores’ volume. Initially opting not to undergo immediate treatment, Patient D was enrolled in AS, which included routine PSA monitoring. A follow up MRI was performed 710 days after diagnosis MRI, and finally, as clear growth was observed, he underwent radical prostatectomy surgery.

Patients E and F are presented in the Supplementary Material file. All six patients share the experience of undergoing AS as their principal treatment strategy. They each have GS of 6 (3 + 3), have been subject to two or more MRI scans, and have had their PSA levels measured in blood tests before and after these imaging procedures. PSA levels exhibit significant variability, yet their trajectory can be accurately characterized by an exponential curve (Karnes et al., 2018; Siebinga et al., 2024) (Figures 4, 5). In this work, two patients were chosen to fine-tune the model parameters for accurate tumour prediction (Patient A and B), while another four were utilized to validate the model’s outcomes (Patient C, D, E, and F). A mesh refinement study was performed and, to yield a good trade off between computational efficiency and accuracy, a mesh of 17833 linear tetrahedral elements and 3829 nodes was identified for the patient A, 20,572 elements and 4274 nodes for patient B, 9185 elements and 2079 nodes for patient C and 18,262 elements and 3900 nodes for patient D. Patient E was meshed with 8804 elements and 1997 nodes and patient F with 55715 elements 10848 nodes.

### 3.2 Optimization results

To perform the optimization presented in Section 2.5, patients A and B are selected.

For the optimization of the growth model, 60 trials are conducted, wherein parameters are adjusted iteratively for each patient to minimize the error function (Supplementary Figure S1A). The Optuna optimizer identifies the best trials based on those that achieve the lowest error. In this case, the optimizer did not find a single set of parameters that minimized the error for both patients simultaneously. Therefore the trial that gives an error of 0.0887 for patient A and 0.108 is selected (further information is provided in the Supplementary Material). Parameter k emerges as the most influential, particularly impactful for patient B. For patient A, both k and $k_{g}$ exhibit significant relevance in model performance, whereas the stiffness of the environment and $k_{v2}$ demonstrate comparatively lesser effects on the overall model ensemble (Supplementary Figure S1B).

For the optimization of the parameters of PSA dynamics, 30 tests are conducted, wherein parameters are adjusted iteratively for each patient to minimize the error function (Supplementary Figure S2A). The minimal absolute error obtained is 0.85 ng/mL for patient A and 0.82 ng/mL for patient B. Parameter γ emerges as the most influential, representing the decay of tissue PSA. The production of PSA of healthy cells is also significantly important $\left(\alpha^{h}\right)$ , followed by the production of tumoral cells $\left(\alpha^{t}\right)$ and the serum PSA decay in blood $\left(\gamma_{s}\right)$ (Supplementary Figure S3).

**FIGURE 6:**
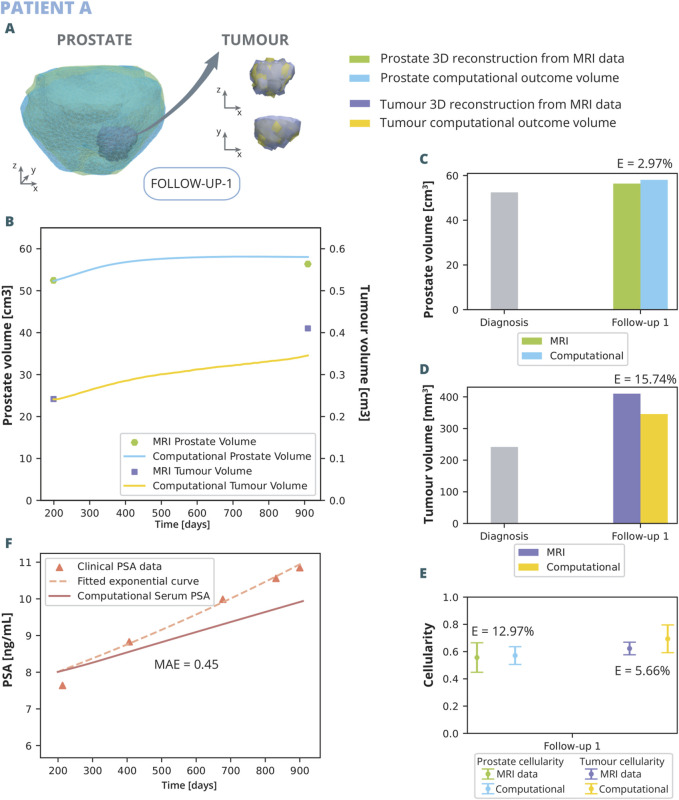
Patient A optimization results: **(A)** shows the simulated geometries of the prostate and the tumour compared to the MRI ones. In **(B)** the growth of the prostate and tumour volume are represented and compared to the MRI segmented volumes. In **(C)** and **(D)**, these volumes have been represented with a bar chart for follow up 1 in order to make a clearer comparison, for the prostate and tumour, respectively. **(E)** represents the overall cellularity in the prostate observed in MRI as opposed to computational outcome. Beside, the cellularity in the tumour area is shown, also comparing the MRI data and computational outcome. Finally, in **(F)**, the simulated serum PSA is compared to the clinical observations.

**FIGURE 7:**
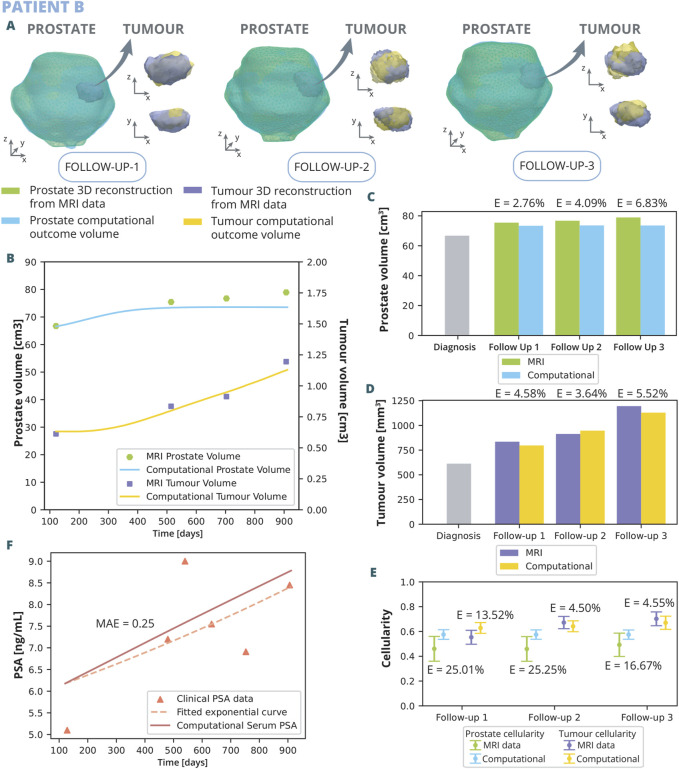
Patient B optimization results: **(A)** shows the simulated geometries of the prostate and the tumour compared to the MRI ones. In **(B)** the growth of the prostate and tumour volume are represented and compared to the MRI segmented volumes. In **(C)** and **(D)**, these volumes have been represented with a bar chart for each follow up in order to make a clearer comparison, for the prostate and tumour, respectively. **(E)** represents the overall cellularity in the prostate observed in MRI as opposed to computational outcome. Beside, the cellularity in the tumour area is shown, also comparing the MRI data and computational outcome. Finally, in **(F)**, the simulated serum PSA is compared to the clinical observations.

The parameters optimised for the model are those listed in Table 1. The results of the optimization, achieved with this specific combination of parameters that yield a better fit, are illustrated in Figures 6, 7.

The outcomes of the computational analysis applied to patient A are described. These outcomes include the geometry of the prostate and tumour compared to one obtained from the MRI data (Figure 6A), as well as graphical representations (Figures 6B–F). Regarding volume growth the simulated prostate and tumour volumes are compared to the segmented from MRI (Figure 6B). The simulated prostate exhibits an initial rapid growth within the initial days, followed by a stabilization phase, ultimately attaining a volume of 55.14 $cm^{3}$ by the first follow up. This closely approximates the MRI observed volume of 56.36 $cm^{3}$ at the corresponding time point with a relative error of 2.97% (Figure 6C). Similarly, the simulated tumour volume experiences an initial rapid increase over the first two hundred days, after which the growth rate decelerates. By the time of the first follow up, the simulated tumour volume reaches 358.36 $mm^{3}$ , in close agreement with the MRI observed volume of 410.15 $mm^{3}$ . This translates into a relative error of 15.74% (Figure 6D). Moreover, the overall cellularity in the prostate and the tumour obtained computationally is compared to that observed clinically (Figure 6E). The numerical outcome demonstrate a commendable alignment with clinical observations, with a relative error of 12.97% in the prostate and 5.66% in the tumour. The mean cellularity in the entire prostate, both clinically and computationally, hovers around 50%, while in the tumour area, it increases to approximately 60%. Even so, this correlation is rationalized by the nature of the healthy prostate, characterized as glandular tissue with numerous lumens. As tumour cells proliferate, these lumens are occupied, resulting in an elevated concentration within the tumour region. As for the PSA dynamics observed (Figure 6F), while the computational curve exhibits a slower rate of growth compared to the clinical curve, the MAE achieved is relatively low, at 0.45 ng/mL. This indicates that, despite the differences in growth rates, the computational model maintains a close approximation to the clinical data.

Patient B underwent three follow up MRI scans prior to radical prostatectomy, which provided a valuable additional insight into the dynamics of cancer growth. As for patient A, the outcomes of the computational analysis applied to patient B are described. These outcomes include the geometry of the prostate and tumour compared to one obtained from the MRI data for each follow-up time point (Figure 7A), as well as graphical representations (Figures 7B–F). Regarding volume growth the simulated prostate and tumour volumes are compared to the ones segmented from MRI (Figure 7B). Similar to the scenario with patient A, the prostate volume for patient B experiences a more rapid growth approximately in the initial 300 days, eventually reaching a plateau by the first follow up. This growth pattern aligns with the observed MRI volumes, emphasizing substantial enlargement from the diagnostic image to follow up 1, obtaining relative errors of 2.76%, 4.09%, and 6.83% for follow up 1, 2, and 3, respectively. In contrast, the growth from follow up 1 to 3 exhibits a less pronounced trend. The evolution of tumour growth follows a sigmoidal pattern, characterized by gradual expansion in the initial phase, acceleration around day 350, and subsequent stabilization around day 800. This trend agrees with MRI data observations, reflecting an 70% increase in tumour size by follow-up 3 compared to its original dimensions, obtaining relative errors of 4.58%, 3.64%, and 5.52% for follow up 1, 2, and 3, respectively. Regarding cellularity (Figure 7E), MRI data observations reveal a relatively stable cellularity in the prostate across the clinical history, whereas the tumorous region experiences a gradual increase over time. Similarly, computational results depict a stabilized cellularity across the prostate, albeit with a lesser degree of variability, achieving relative errors of 25.01%, 25.25%, and 16.67% for follow up 1, 2, and 3, respectively. Notably, the cellularity in the computational tumour exhibits a more restrained growth compared to its MRI data counterpart. The results yielded relative errors of 13.52% for the follow up 1, 4.50% for the follow up 2, and 4.55% for the follow up 3. The dynamics of clinical and serum PSA are also depicted (Figure 7F). In this simulation, the PSA levels increased more rapidly than the exponential curve fitting the clinical data. Nevertheless, a MAE of 0.25 ng/mL was achieved.

### 3.3 Validation of the model

**FIGURE 8:**
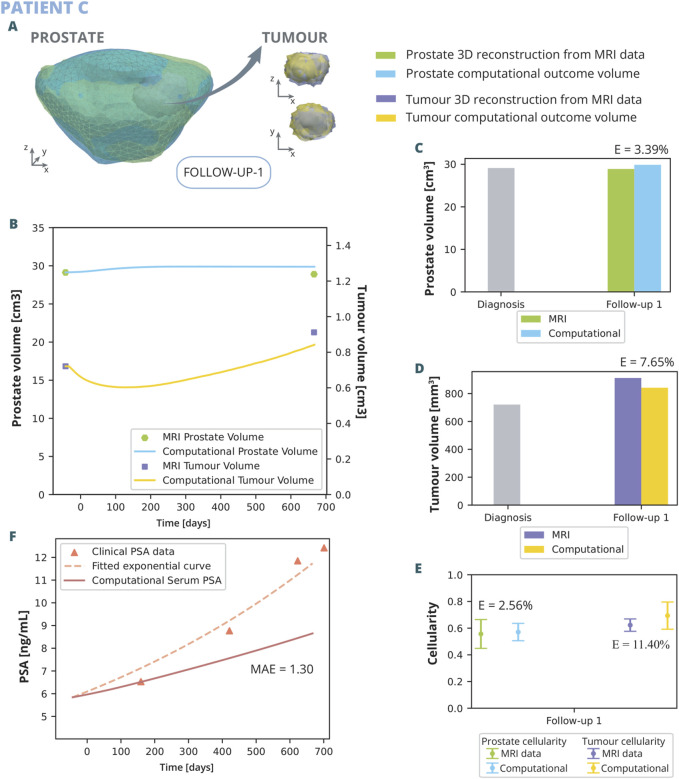
Patient C simulation results: **(A)** shows the simulated geometries of the prostate and the tumour compared to the MRI ones. In **(B)** the growth of the prostate and tumour volume are represented and compared to the MRI segmented volumes. In **(C)** and **(D)**, these volumes have been represented with a bar chart for follow up 1 in order to make a clearer comparison, for the prostate and tumour, respectively. **(E)** represents the overall cellularity in the prostate observed in MRI as opposed to computational outcome. Beside, the cellularity in the tumour area is shown, also comparing the MRI data and computational outcome. Finally, in **(F)**, the simulated serum PSA is compared to the clinical observations.

**FIGURE 9:**
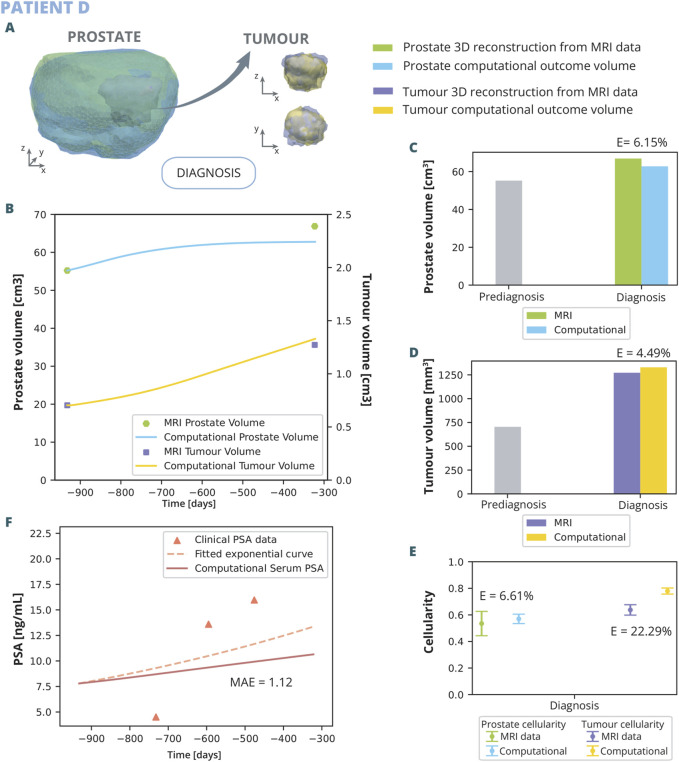
Patient D simulation results: **(A)** shows the simulated geometries of the prostate and the tumour compared to the MRI ones. In **(B)** the growth of the prostate and tumour volume are represented and compared to the MRI segmented volumes. In **(C)** and **(D)**, these volumes have been represented with a bar chart for follow up 1 in order to make a clearer comparison, for the prostate and tumour, respectively. **(E)** represents the overall cellularity in the prostate observed in MRI as opposed to computational outcome. Beside, the cellularity in the tumour area is shown, also comparing the MRI data and computational outcome. Finally, in **(F)**, the simulated serum PSA is compared to the clinical observations.

The results of the application of the model to the patients outlined in the previous section are here presented (Figures 8, 9).

The outcomes of the computational analysis applied to patient C are described following the same pattern as in previous subsection. These outcomes include the geometry of the prostate and tumour compared to one obtained from the MRI data (Figure 8A), as well as graphical representations (Figures 8B–F). Regarding volume growth the simulated prostate and tumour volumes are compared to the clinical ones (Figure 8B). The MRI prostate volume exhibits no significant increase, whereas the simulated volume demonstrates a slight initial growth before quickly stabilizing, resulting in a relative error of 3.39% during the first follow-up (Figure 8C). Conversely, the simulated tumour volume displays an initial decrease, followed by a rapid increase, eventually aligning with the MRI tumour volume with a relative error of 7.65% (Figure 8D). Additionally, the cellularity within the prostate and the tumour, as determined computationally, is corroborated by MRI data observations (Figure 8E). The numerical analysis yields a close approximation to the empirical data, with a relative error of 2.56% for the prostate and 11.40% for the tumour. A higher variability in the simulated cellularity is noted for the tumour compared to that detected in MRI. Regarding the observed PSA dynamics (Figure 8F), the computational model delineates a more gradual increase in PSA levels when contrasted with the clinical trajectory, evidenced by a MAE of 1.3 ng/mL. The model demonstrates limitations in replicating the swift escalation observed in PSA concentrations.

The outcomes of the computational analysis applied to patient D are described. These outcomes include the geometry of the prostate and tumour compared to one obtained from the MRI data (Figure 8A), as well as graphical representations (Figures 8B–F). Regarding volume growth the simulated prostate and tumour volumes are compared to the clinical ones (Figure 8B). In the computational simulations, an initial rapid increase in prostate volume is noted, which subsequently stabilizes. Upon reaching the date of the first MRI follow-up, the simulated volume attains 62.76 $cm^{3}$ . This represents a relative error of 6.15% when compared to the volume segmented from the MRI data (Figure 8C). Conversely, the tumour exhibits a notably accelerated expansion, with MRI segmentations indicating a growth rate of 80.71%. The computational model closely mirrors this progression, achieving a relative error of just 4.49% at the time of the first follow-up. Moreover, the cellularity metrics for the prostate and the tumour, as inferred from computational models, are validated against the MRI data observations (Figure 8E). The computational analysis presents a proximate reflection of the actual data, with a relative error of 6.61% for the prostate and 22.29% for the tumour. Notably, this iteration reveals a more pronounced variance between the simulated cellularity and that observed in MRI. Concerning the PSA dynamics depicted (Figure 8F), the computational model continues to project a more subdued ascent in PSA levels compared to the clinical progression, as indicated by a MAE of 1.12 ng/mL. This discrepancy underscores the model’s challenge in capturing the rapid surge in PSA levels that is evident in clinical observations.

Results of Patient E and F are shown in the Supplementary Material. Similar trends on the predictions were obtained for these two additional patients.

## 4 Discussion

In this work, a novel approach is presented for creating a PCa model by integrating clinical MRI data, so a full representation of the whole prostate is obtained with its cellular and vascular characteristics and the location of the tumour. On top of the personalized model construction, our approach simulates and predicts the effect of tumour growth in the prostate through the FE method, coupling the dynamics of growth and the transport of oxygen to better simulate the cross-talk between cells and tumour microenvironment (Rao (2011); Ambrosi et al. (2019); Comellas et al. (2018); Hervas-Raluy et al. (2024)). In addition, our approach includes the simulation of the PSA dynamics, which allows to evaluate tumour growth through the PSA levels in the patient, that is a standard-of-care during AS.

The parameters of the biomechanical model presented here have been predominantly gathered from existing literature, yet refinement through optimization has been also performed to adjust the model. It is assumed that the mechanisms of cell proliferation and cell death are activated according to the oxygen concentration: adequate oxygen triggers proliferation, while its absence induces cell death. The model can be expanded to integrate additional factors such as glucose or other nutrients. However, due to the complexity of modeling all mechanisms involved in tumour growth, oxygen has been selected as a representative factor for all these nutrients. Future models could incorporate such additional elements by modifying the transport equations to examine their impact on tumour growth dynamics. Furthermore, the model incorporates a widely-recognized regulatory term that reduces the rate of proliferation as cell density approaches the tissue’s carrying capacity, ensuring that cell densities remain within biologically plausible limits. This term is essential for modulating the model’s growth dynamics, as demonstrated by the optimization analysis detailed in the Supplementary Material. The assumed carrying capacity of tumour cells surpasses that of healthy cells, accounting for the glandular structure in healthy tissue. Proliferation rates are further influenced by the level of cell differentiation, with more undifferentiated tumour cells exhibiting higher growth rates. The proliferation rate parameter significantly impacts growth dynamics, especially in patient A, where it ranks as the second most important factor according to the optimization analysis (see Supplementary Material). In this model, the diffusion term is underestimated from the equations that govern population density evolution. The focus is on predicting tumour growth in a localized context during the initial stages of cancer. Consequently, for simplicity, the assumption is that the cancer has not yet metastasized (Cieślikowski et al., 2020; Klotz, 2020). This simplification is a significant limitation, as it prevents the model from simulating tumour infiltration into surrounding tissues.

The distribution of oxygen within the tumour is determined by a mass transport model. To mitigate computational costs, two assumptions are incorporated. Firstly, the diffusive process of the species is considered negligible compared to the extravasation and consumption terms (Pienta and Loberg, 2005). And secondly, recognising the substantial difference in time scales between cellular processes and transport phenomena, both models are integrated into a multiscale temporal model.

In the context of PSA dynamics, a temporal model is also taken into account, postulating the attainment of equilibrium between PSA concentration in the tissue and the blood before the conclusion of each time increment. The computation of PSA in the tissue involves considering that in the tumour area, where lumens and ducts are obstructed due to abnormal cell proliferation, the accumulation of PSA in the tissue surpasses that in the healthy region (Lilja et al., 2008; Djavan et al., 1999). To account for this effect, the proliferation range of healthy cells is chosen to be much lower than that of tumour cells. Consequently, this results in a parameter for healthy cell production that is 120 times lower in our model’s calibration. It’s important to note that this parameter falls outside the typical biological range, and as such, should be interpreted with caution. With regard to natural decay rates, it is noteworthy that although the natural decay rates observed during optimization appear to deviate from the expected natural ranges, discrepancies were also observed in other models (Jain et al., 2011), so there is no clear consensus. This discrepancy highlights the inherent challenges in identifying parameters that fit the model seamlessly. Such difficulties are not uncommon and reflect the complex nature of accurately simulating real world phenomena. The production rates of PSA are critical to this dynamic, particularly those from healthy cells, constitute a greater portion of the total prostate volume, despite the fact that PSA leakage into the tissue is more intense in the tumour zone. The most significant parameter in PSA dynamics, however, is the tissue decay rate (see Supplementary Material). Moreover, vascularization is acknowledged as the primary facilitator of the exchange between PSA in the tissue and blood (Chung et al., 2014; Wu et al., 2018). The key parameter that conveys information about prostate vascularisation is $K^{\mathrm{Trans}}$ , influencing the arrival of oxygen to the tissue and the exchange between PSA in the tissue and blood. Other previous prostate tumour growth models (Lorenzo et al., 2024; Jain et al., 2011) neglects this valuable patient-specific information. A limitation of the present work is that $K^{\mathrm{Trans}}$ is treated as static, deviating from reality. Recognising the importance of angiogenesis in tumour growth, future efforts should explore the implementation of a dynamic $K^{\mathrm{Trans}}$ for a more accurate representation. In line with earlier models (Jain et al., 2011), diffusion is neglected from PSA dynamics equations. We assume the convective transport is much more significant compared to the slower and limited impact of diffusion. The quick changes in PSA levels and the small differences in concentration further lessen the importance of diffusion (Pienta and Loberg, 2005; Tartakovsky and Dentz, 2019; Hervas-Raluy et al., 2024).

PSA is the main biomarker currently used by clinicians to assess the progression of cancer during AS, hence it seems likely that a rise in PSA is related to the progression of the disease. However, clinical PSA measurements exhibit substantial variability, as PSA levels can significantly fluctuate due to various factors, whether intrinsic to the patient -such as elevations due to benign prostatic hypertrophy, recent manipulation of the prostate due to massage or biopsy or prostatitis (Pezaro et al., 2014; Tzelepi 2023; Maeda-Minami et al., 2023)- or external. In fact, PSA variability is significant in PCa diagnostics, with studies linking fluctuations to increased cancer risk (Maeda-Minami et al., 2023). Public databases provide insights into PSA patterns in both non-PCa and PCa patients (Karunasinghe et al., 2022). PSA levels, influenced by various factors, vary with cancer stage and are not strict cutoffs. Higher PSA levels generally indicate advanced disease, but clinical stage and Gleason Score also stratify risk (Tzelepi, 2023). Despite these variations, the trajectory of the PSA data can be accurately characterized by an exponential curve (Karnes et al., 2018; Siebinga et al., 2024) (Figures 4, 5).

In this paper, two patients are used for parameter optimization of the model (patient A and B) and four others for demonstrating the preliminary feasibility of the model (patient C, D, E and F). The four six patients initially pursued AS as the primary treatment approach, involving multiple PSA measurements and MRI scans. Despite the presence of extraprostatic extension in patient B, the inclusion of patient B in the study was deemed necessary due to the challenges associated with obtaining new patient data. Moreover, given that the model is designed to simulate the tumour within a defined boundary, it remains applicable for its intended purpose. By integrating patient-specific data obtained from MRIs and biopsies, the model accounts for inter-patient variability. This approach avoids the need for individual patient data calibration, favoring a unified parameter set applicable across the patient spectrum. Consequently, it facilitates personalized predictions that are both efficient and tailored to the input data, thereby optimizing the predictive process. Finding suitable parameters for biological models is a complex endeavor. The intricate nature of biological systems means that small changes in parameters can lead to significant differences in outcomes, making it challenging to align models with real-world data. This difficulty is a notable barrier in the development of accurate predictive models. Despite the limited number of validated cases, the computationally results obtained closely align with the clinically observed outcomes. To gain a deeper understanding of the various mechanisms involved in tumour development, it would be essential to access additional data, which are currently unavailable. It is noteworthy to emphasize the difficulty in obtaining comprehensive datasets for analyses of this nature. These are retrospective studies of patients treated at the hospital, where prioritizing minimal and non-invasive tests is imperative, ensuring the patient’s health and comfort take precedence. In regards to cellularity, the overall prostate cellularity is stable over time. In contrast, tumour cellularity increases. This variance is rationalised by the stable cell density and structural maintenance in the healthy tissue, which contrasts with the abnormal cell proliferation in the tumour tissue. In the computational results we obtain a lower dispersion for both cases, but the mean aligns within the clinical observations. The reason for this is that the model inherently promotes uniformity in prostate cellularity, representing a discernible constraint. Advancements in the model’s design and further refinement will be necessary to better mimic cellularity outcome. It would indeed be advantageous to have the capability to replicate cellular distribution by modifying or introducing new hypotheses. This would enhance the model’s adaptability and accuracy in reflecting the complex biological processes within the prostate. Moreover, the complexities involved in accurately quantifying discrepancies between the cellularity maps from the model and actual observations are acknowledged. These challenges arise from the absence of direct correspondence between the mesh from the simulated prostate and the mesh generated from MRI in the follow ups, which remain unregistered and vary in their nodes and elements. Consequently, the focus has been maintained on the primary objective of the work: to simulate tumour growth, prostate, and PSA dynamics.

It is crucial to emphasize that the data used may harbor intrinsic errors originating from image acquisition procedures and assumptions made during data preprocessing. The volumes of both the tumour and prostate are susceptible to dimensional errors stemming from various factors. These include inaccuracies in segmentation, an insufficient number of MRI slices to capture the 3D characteristics, leading to potential inaccuracies in 3D reconstruction, smoothing procedures to rectify imperfections in the reconstruction, and the meshing of the geometry. Despite these challenges, our model serves as a valuable tool for simulating and understanding key aspects of PCa dynamics, providing a foundation for further refinement and improvement in the pursuit of enhanced accuracy and predictive capabilities.

This work represents a first contribution to the development of a future digital PCa twin. Future directions include the integration of different cancer treatments such as RT and HT into the model to study the effects of these treatments on tumour growth. The core vision for the digital twin of PCa is to predict the various treatment scenarios a newly diagnosed patient might face, such as AS, HT, RT, and others. This type of model holds the potential to significantly advance clinical practice. In the future, patient-specific digital twins for PCa could become a valuable tool, enabling clinicians to predict how the disease will progress in a patient-specific manner. This would aid in determining the most appropriate treatment plan, reducing the risks of both over- and under-treatment, which can cause considerable distress, improving that way the quality of life of the patient. The development of digital twins aligns with the principles of precision medicine, marking a paradigm shift in the approach to clinical decisions for PCa patients and paving the way for more effective and targeted interventions in cancer care.

## Data Availability

The raw data supporting the conclusions of this article will be made available by the authors, without undue reservation.
