# Supplementary material for: Patient-specific prostate tumour growth simulation: a first step towards the digital twin
Source: Front Physiol. 2024 Oct 30;15:1421591. doi: 10.3389/fphys.2024.1421591 (PMC11557540; doi:10.3389/fphys.2024.1421591)
Supplement: Supplementary file 1 [file Datasheet1.pdf]

Supplementary Material

1 IMAGING AQUISITION PARAMETERS

Table S1 and S2 contain detailed information on the acquisition parameters of the MRIs.

2 FURTHER OPTIMIZATION RESULTS

For the optimization of the growth model, 60 trials are conducted, wherein parameters are adjusted iteratively for each patient to minimize the error function (Fig. S1 (A)). Furthermore, the significance of the four parameters subject to optimization is highlighted (Fig. S1 (B)). Parameter k emerges as the most influential, particularly impactful for patient B. For patient A, both k and k_g exhibit significant relevance in model performance, whereas the stiffness of the environment and k_{v2} demonstrate comparatively lesser effects on the overall model ensemble. The Optuna optimizer determines the optimal trials by identifying those with the lowest error. In this case, as a multiple optimization was performed involving two patients, it was not possible for the optimizer to identify a unified parameter set that simultaneously minimized the error for both. Table 4 summarizes the best trials along with their respective relative errors. Out of all the top-performing trials (Fig. S3), Best Trial 1 and Best Trial 3 are the ones that most accurately represent the growth dynamics. The values represented are close enough. Finally, parameters from Best Trial 1 were selected as the final set, given the close similarity of results across the top trials and necessitating the choice of a singular parameter set.

For the optimization of the parameters of PSA dynamics, 30 trials are conducted, wherein parameters are adjusted iteratively for each patient to minimize the error function (Fig. S2 (A)). The minimal absolute error obtained is 0.85 ng/mL for patient A and 0.82 ng/mL for patient B. Furthermore, the significance of the four parameters subject to optimization is highlighted (Fig. S2 (B)). Parameter γ emerges as the most influential, representing the decay of tissue PSA. The production of PSA of healthy cells is also significantly important (α^h), followed by the production of tumoral cells (α^t) and the serum PSA decay in blood (γ_s).

3 FURTHER VALIDATION RESULTS

In this section, the outcome of two more patient case are predicted in order to further validate the model presented.

Patient E (Fig.S4) was diagnosed at the age of 66. A review of his medical history revealed no personal or family history of cancer. An MRI performed 373 days before the biopsy identified a PI-RADS 4 lesion in the medial portion of the peripheral zone, spanning the mid and apex regions. The biopsy showed a GS of 6 (3+3), with tumor involvement accounting for an average of 0.5% of the sampled core volume. Initially, Patient E was enrolled AS with regular PSA monitoring instead of immediate treatment. A follow-up MRI, conducted 322 days after the diagnosis, showed significant tumor growth, leading to RT.

Patient F (Fig.S4) was diagnosed at 62 years old, with no personal or family history of cancer. An MRI conducted 763 days after the initial biopsy identified a PI-RADS 4 lesion in the medial peripheral zone, extending through the mid and apex regions. The biopsy revealed a GS of 6 (3+3), with tumor involvement

averaging 1.05% of the sampled core volume. Patient F initially was registered for AS, involving regular PSA monitoring, rather than immediate treatment. Despite a follow-up MRI 1253 days after diagnosis showing notable tumor growth, AS remained the preferred management approach.

Patient E was meshed with 8804 elements and 1997 nodes and patient F with 55715 elements 10848 nodes.

The outcomes of the computational analysis applied to patient E are described. These outcomes include the geometry of the prostate and tumour compared to one obtained from the MRI data (Fig.S5 (A)), as well as graphical representations (Fig.S5 (B, C, D, E, F)). The simulated prostate and tumor volumes were compared to the clinical data (Fig.S5 (B)) to assess volume growth. In the computational simulations, there was an initial rapid increase in prostate volume, which later stabilized. By the time of the first MRI follow-up, the simulated prostate volume reached 31.30 cm^3, representing a relative error of 3.76% compared to the MRI-segmented volume (Fig.S5 (C)). In contrast, the tumor showed a significantly faster growth rate, and the computational model closely matched this trend, with a relative error of only 20.29% at the first follow-up. Additionally, cellularity metrics for both the prostate and the tumor, derived from the computational models, were compared to MRI observations (Fig.S5 (E)). The analysis yielded relatively accurate results, with a 19.14% error for the prostate and just 1.52% for the tumor, though there was a more noticeable discrepancy in the simulated cellularity for the prostate compared to MRI data. In terms of PSA dynamics (Fig.S5 (F)), the computational model predicted a more rapidly increase in PSA levels than observed clinically. However, it adjusts with the clinial data, obtaining a MAE of 0.37 ng/mL.

The outcomes of the computational analysis applied to patient F are described following the same pattern as in previous subsection. These outcomes include the geometry of the prostate and tumour compared to one obtained from the MRI data (Fig.S6 (A)), as well as graphical representations (Fig.S6 (B, C, D, E, F)). Regarding volume growth the simulated prostate and tumour volumes are compared to the clinical ones (Fig.S6 (B)). The MRI prostate volume exhibits an slight increase, whereas the simulated volume demonstrates a slight initial growth before quickly stabilizing, resulting in a relative error of 4.12% during the first follow-up (Fig.S6 (C)). Conversely, the tumor showed a significantly faster growth rate, and the computational model closely matched this trend, eventually aligning with the MRI tumour volume with a relative error of 13.02% (Fig.S6 (D)). Additionally, the cellularity within the prostate and the tumour, as determined computationally, is corroborated by MRI data observations (Fig.S6 (E)). The numerical analysis yields a close approximation to the empirical data, with a relative error of 6.33% for the prostate and 7.31% for the tumour. A higher variability in the simulated cellularity is noted for the tumour compared to that detected in MRI. Regarding the observed PSA dynamics (Fig.S6 (F)), the computational model closely alings with the fitted exponential curve and the clinical PSA data, evidenced by a MAE of 0.37 ng/mL.

4 SUPPLEMENTARY TABLES AND FIGURES

		PATIENT A		PATIENT B		
		DIAGNOSIS	FOLLOW-UP-1	DIAGNOSIS	FOLLOW-UP-1	FOLLOW-UP-2
T2w	**Manufacturer**	GE MEDICAL SYSTEMS	GE MEDICAL SYSTEMS	GE MEDICAL SYSTEMS	GE MEDICAL SYSTEMS	GE MEDICAL SYSTEMS
	**Manufacturer Model**	Signa HDxt	Signa HDxt	Signa HDxt	Signa HDxt	Signa HDxt
	**Magnetic field Strength [T]**	3	3	3	3	3
	**RepetitionTime [ms]**	6520	6480	4680	2740	2740
	**EchoTime [ms]**	91.2	92.568	148.896	90.896	90.896
	**SliceThickness [mm]**	3	3	3	3	3
	**Spacing Between Slices [mm]**	3	3	3.3	3	3
	**AcquisitionMatrix**	[320, 0, 0, 224]	[320, 0, 0, 224]	[320, 0, 0, 256]	[320, 0, 0, 224]	[320, 0, 0, 224]
	**PixelSpacing [mm]**	[0.3125, 0.3125]	[0.3125, 0.3125]	[0.3516, 0.3516]	[0.3125, 0.3125]	[0.3125, 0.3125]
DWI	**Manufacturer**	GE MEDICAL SYSTEMS	GE MEDICAL SYSTEMS	GE MEDICAL SYSTEMS	GE MEDICAL SYSTEMS	GE MEDICAL SYSTEMS
	**Manufacturer Model**	Signa HDxt	Signa HDxt	Signa HDxt	Signa HDxt	Signa HDxt
	**Magnetic field Strength [T]**	3	3	3	3	3
	**RepetitionTime [ms]**	6000	6000	7000	6000	6000
	**EchoTime [ms]**	75.3	75.4	98	75.4	75.3
	**SliceThickness [mm]**	3	3	3	3	3
	**Spacing Between Slices [mm]**	3	3	3.3	3	3
	**AcquisitionMatrix**	[64, 0, 0, 64]	[64, 0, 0, 64]	[64, 0, 0, 64]	[64, 0, 0, 64]	[64, 0, 0, 64]
	**PixelSpacing [mm]**	[0.625, 0.625]	[0.625, 0.625]	[0.7031, 0.7031]	[0.625, 0.625]	[0.625, 0.625]
	**b-value [s/mm^3]**	0-1000	0-1000	0-1500	0-1000	0-1000
DCE	**Manufacturer**	GE MEDICAL SYSTEMS	GE MEDICAL SYSTEMS	GE MEDICAL SYSTEMS	GE MEDICAL SYSTEMS	GE MEDICAL SYSTEMS
	**Manufacturer Model**	Signa HDxt	Signa HDxt	Signa HDxt	Signa HDxt	Signa HDxt
	**Magnetic field Strength [T]**	3	3	3	3	3
	**RepetitionTime [ms]**	3.644	3.644	4.276	3.636	3.636
	**EchoTime [ms]**	1.676	1.68	2.016	1.672	1.672
	**SliceThickness [mm]**	3	3	4	3	3
	**Spacing Between Slices [mm]**	1.5	1.5	2	1.5	1.5
	**AcquisitionMatrix**	[256, 0, 0, 192]	[256, 0, 0, 192]	[256, 0, 0, 192]	[256, 0, 0, 192]	[256, 0, 0, 192]
	**Pixel Spacing [mm]**	[1.3281, 1.3281]	[1.3281, 1.3281]	[0.5859, 0.5859]	[1.3281, 1.3281]	[1.3281, 1.3281]
	**Number of dynamics**	30	30	30	30	30
	**Acquisition time [s]**	91.311	155.15	122.811	195.65	162.02
	**Time resolution [s]**	3.04	5.17	4.0937	6.52	5.4

Table S1. MRI acquisition parameters

		PATIENT B	PATIENT C		PATIENT D	
		FOLLOW-UP-3	DIAGNOSIS	FOLLOW-UP-1	PREDIAGNOSIS	DIAGNOSIS
T2w	**Manufacturer**	Siemens HealthCare GmbH	GE MEDICAL SYSTEMS	Siemens HealthCare GmbH	GE MEDICAL SYSTEMS	GE MEDICAL SYSTEMS
	**Manufacturer Model**	MAGNETOM Vida	Signa HDxt	MAGNETOM Vida	Signa HDxt	Signa HDxt
	**Magnetic field Strength [T]**	3	3	3	3	3
	**RepetitionTime [ms]**	6920	2140	7750	2760	3760
	**EchoTime [ms]**	107	145.872	107	91.732	93.86
	**SliceThickness [mm]**	3	3	3	3	3
	**Spacing Between Slices [mm]**	3	3.3	3	3	3
	**AcquisitionMatrix**	[0, 384, 307, 0]	[320, 0, 0, 256]	[0, 384, 307, 0]	[320, 0, 0, 224]	[320, 0, 0, 224]
	**PixelSpacing [mm]**	[0.546875, 0.546875]	[0.3516, 0.3516]	[0.546875, 0.546875]	[0.3125, 0.3125]	[0.3125, 0.3125]
DWI	**Manufacturer**	Siemens HealthCare GmbH	GE MEDICAL SYSTEMS	Siemens HealthCare GmbH	GE MEDICAL SYSTEMS	GE MEDICAL SYSTEMS
	**Manufacturer Model**	MAGNETOM Vida	Signa HDxt	MAGNETOM Vida	Signa HDxt	Signa HDxt
	**Magnetic field Strength [T]**	3	3	3	3	3
	**RepetitionTime [ms]**	5180	6000	6500	6000	6500
	**EchoTime [ms]**	58	74.6	92	75.3	75.4
	**SliceThickness [mm]**	3	3	3	3	3
	**Spacing Between Slices [mm]**	3	3.3	3	3	3
	**AcquisitionMatrix**	[134, 0, 0, 70]	[64, 0, 0, 64]	[150, 0, 0, 57]	[64, 0, 0, 64]	[64, 0, 0, 64]
	**PixelSpacing [mm]**	[0.895522, 0.895522]	[0.7031, 0.7031]	[1.63333, 1.63333]'	[0.625, 0.625]	[0.625, 0.625]
	**b-value [s/mm^3]**	50-1400	0-1000	50-1400	0-1000	0-1000
DCE	**Manufacturer**	Siemens HealthCare GmbH	GE MEDICAL SYSTEMS	Siemens HealthCare GmbH	GE MEDICAL SYSTEMS	GE MEDICAL SYSTEMS
	**Manufacturer Model**	MAGNETOM Vida	Signa HDxt	MAGNETOM Vida	Signa HDxt	Signa HDxt
	**Magnetic field Strength [T]**	3	3	3	3	3
	**RepetitionTime [ms]**	3.51	4.248	3.51	3.648	3.68
	**EchoTime [ms]**	1.29	2.008	1.29	1.676	1.684
	**SliceThickness [mm]**	3	4	3	3	3
	**Spacing Between Slices [mm]**	1.5	2	1.5	1.5	1.5
	**AcquisitionMatrix**	[224, 0, 0, 167]	[256, 0, 0, 192]	[224, 0, 0, 167]	[256, 0, 0, 192]	[256, 0, 0, 192]
	**Pixel Spacing [mm]**	[1.16071, 1.16071]	[0.5859, 0.5859]	[1.16071, 1.16071]	[1.3281, 1.3281]	[1.3281, 1.3281]
	**Number of dynamics**	30	30	30	30	30
	**Acquisition time [s]**	84.14	90.336	183.71	212.355	212.528
	**Time resolution [s]**	2.804666667	3.0112	6.123666667	7.0785	7.084266667

Table S2. MRI acquisition parameters

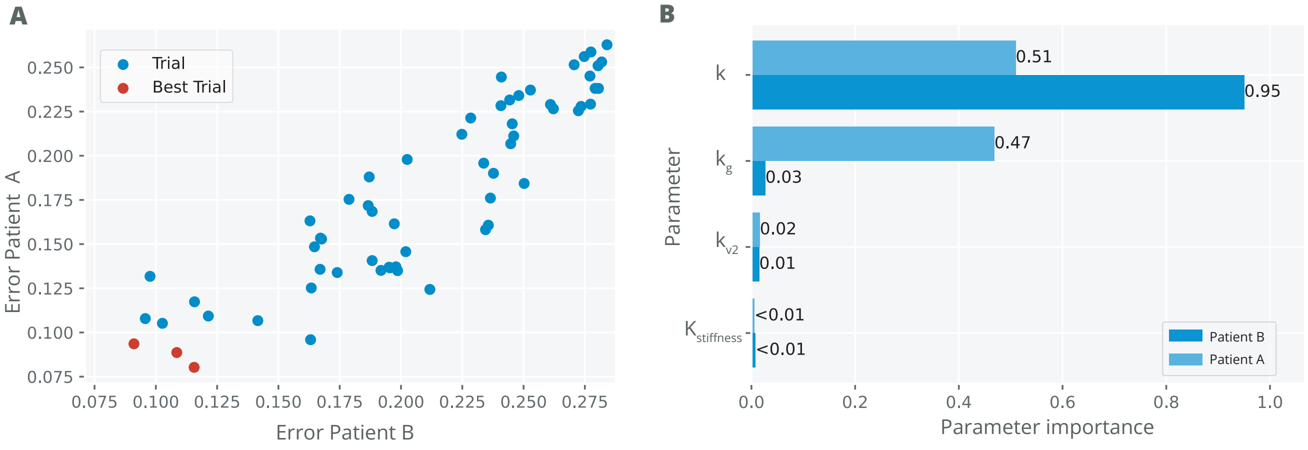

Figure S1. Optimization of the model of prostate cancer. (A) Pareto-front plot (B) Parameter importance.

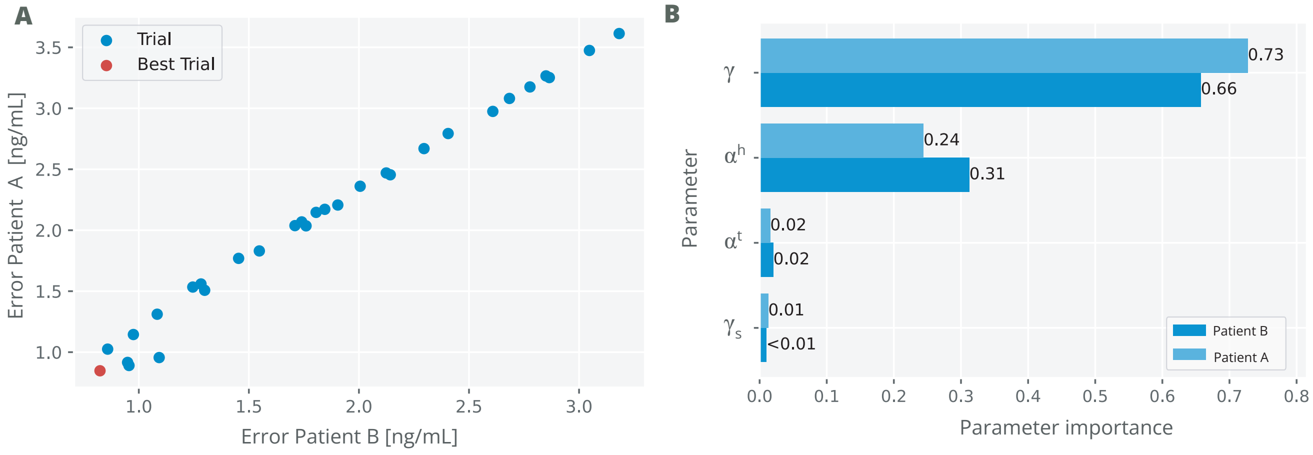

Figure S2. Optimization of the model of PSA dynamics. (A) Pareto-front plot (B) Parameter importance.

	**Patient A**	**Patient B**
Best Trial 1	0.0887	0.108
Best Trial 2	0.093	0.091
Best Trial 3	0.080	0.115

Table S3. Relative errors obtained in the best trials for Patient A and B.

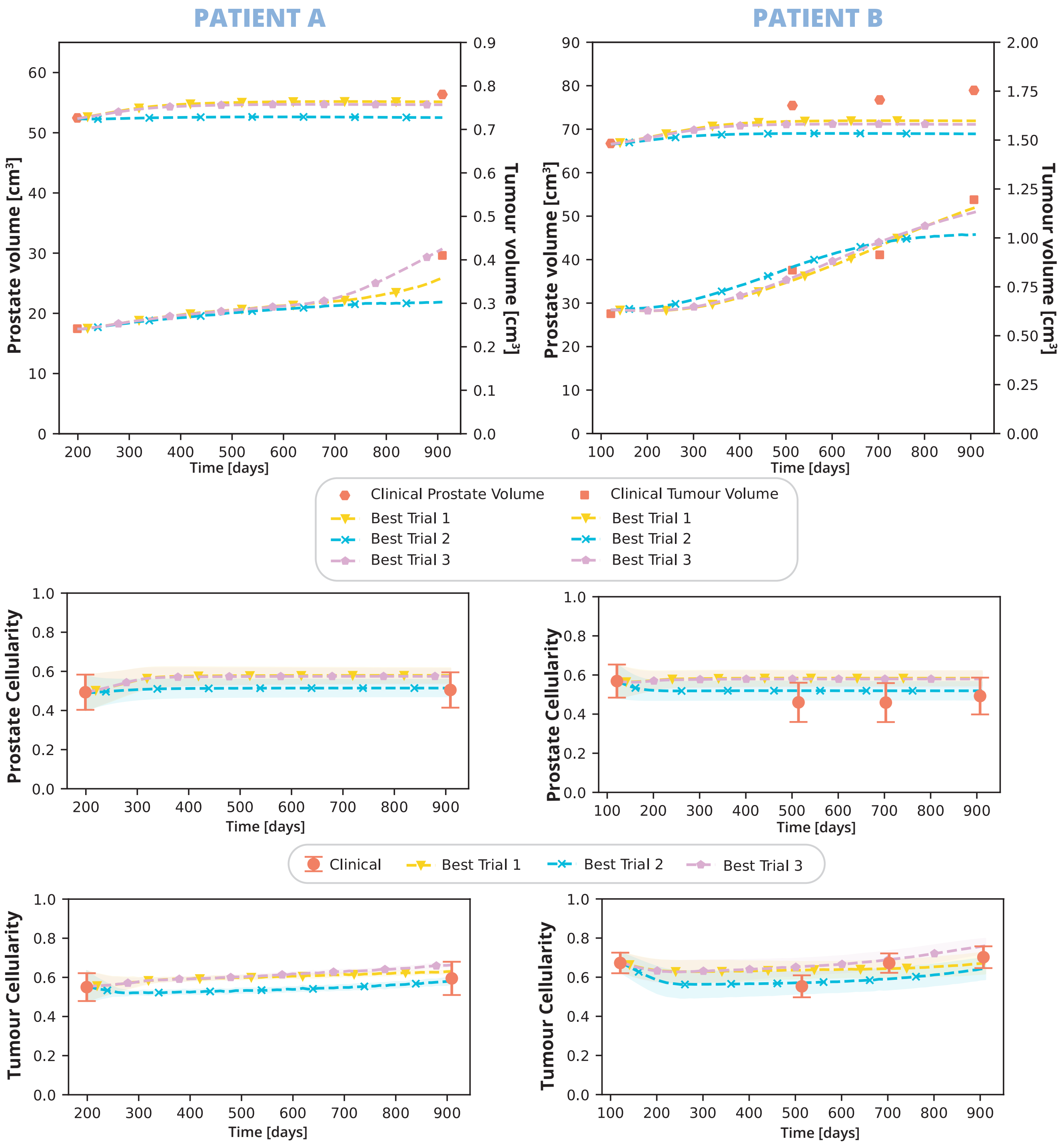

Figure S3. Results of the best trials for both patients: (Top) Growth of prostate and tumor volume. (Middle) Mean prostate cellularity. (Bottom) Mean cellularity within the tumor. All objective values are also represented in orange (Clinical values).

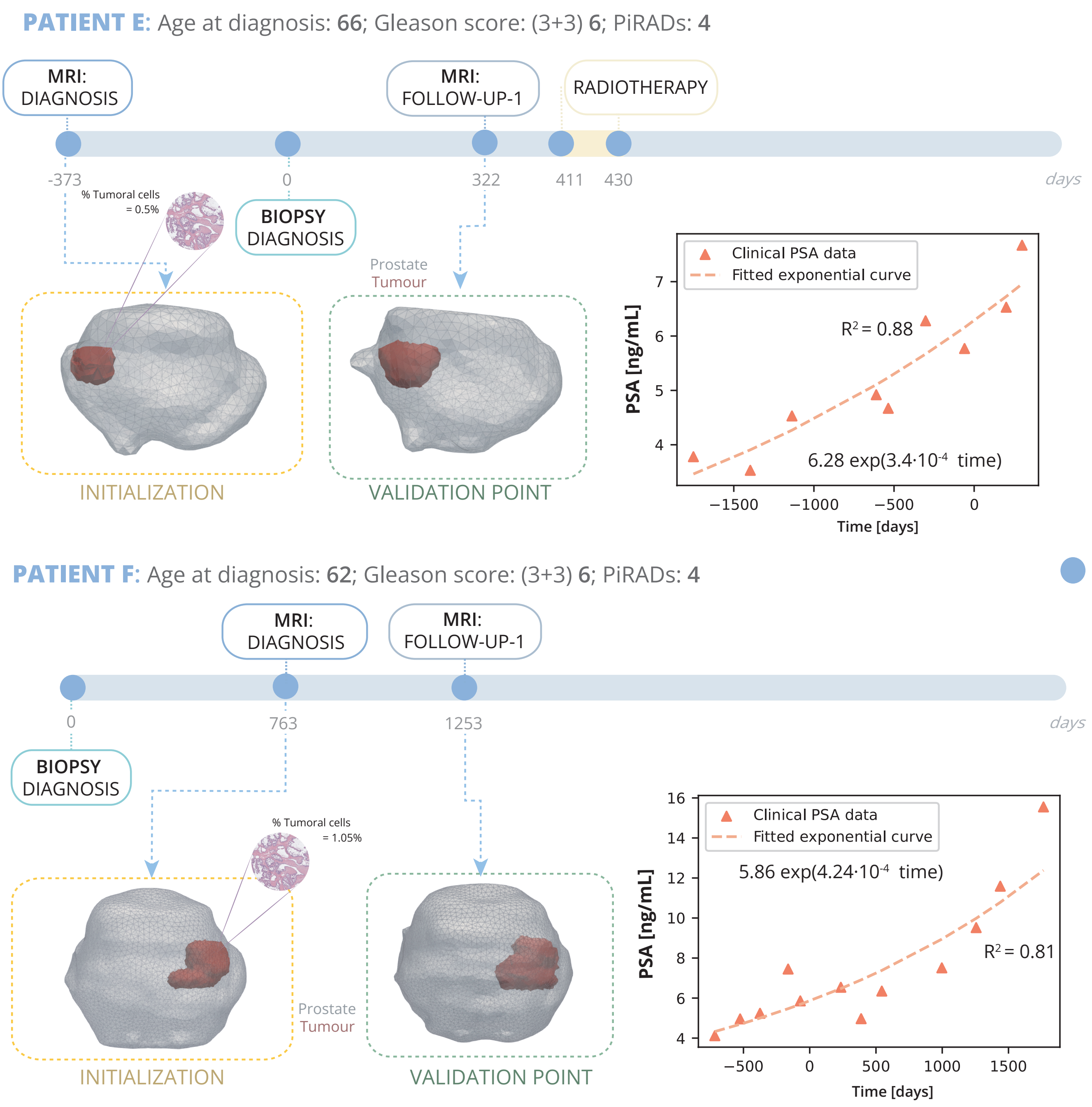

Figure S4. Patient E and F clinical history: Patient E, diagnosed at 66 with grade 4 PI-RADS, had a Gleason Score of 6 (3+3) and 0.5% tumour volume; underwent RT 411 days post-diagnosis following a single follow-up MRI at day 322. Patient F, diagnosed at 62 with grade 4 PI-RADS, had a Gleason Score of 6 (3+3) and 1.05% tumour volume; he underwent 2 MRI, one of diagnosis at day 763 and another for follow-up 1 at day 1253. On the right, PSA measurements for both patients are displayed alongside their fitted exponential growth curves. Bellow the timeline, the FE mesh digital reconstructions of the prostate and tumours at diagnosis and follow-up MRIs is showed. The first MRI taken for each patient are used for the initialization of the model, while the subsequent MRI serves for validation.

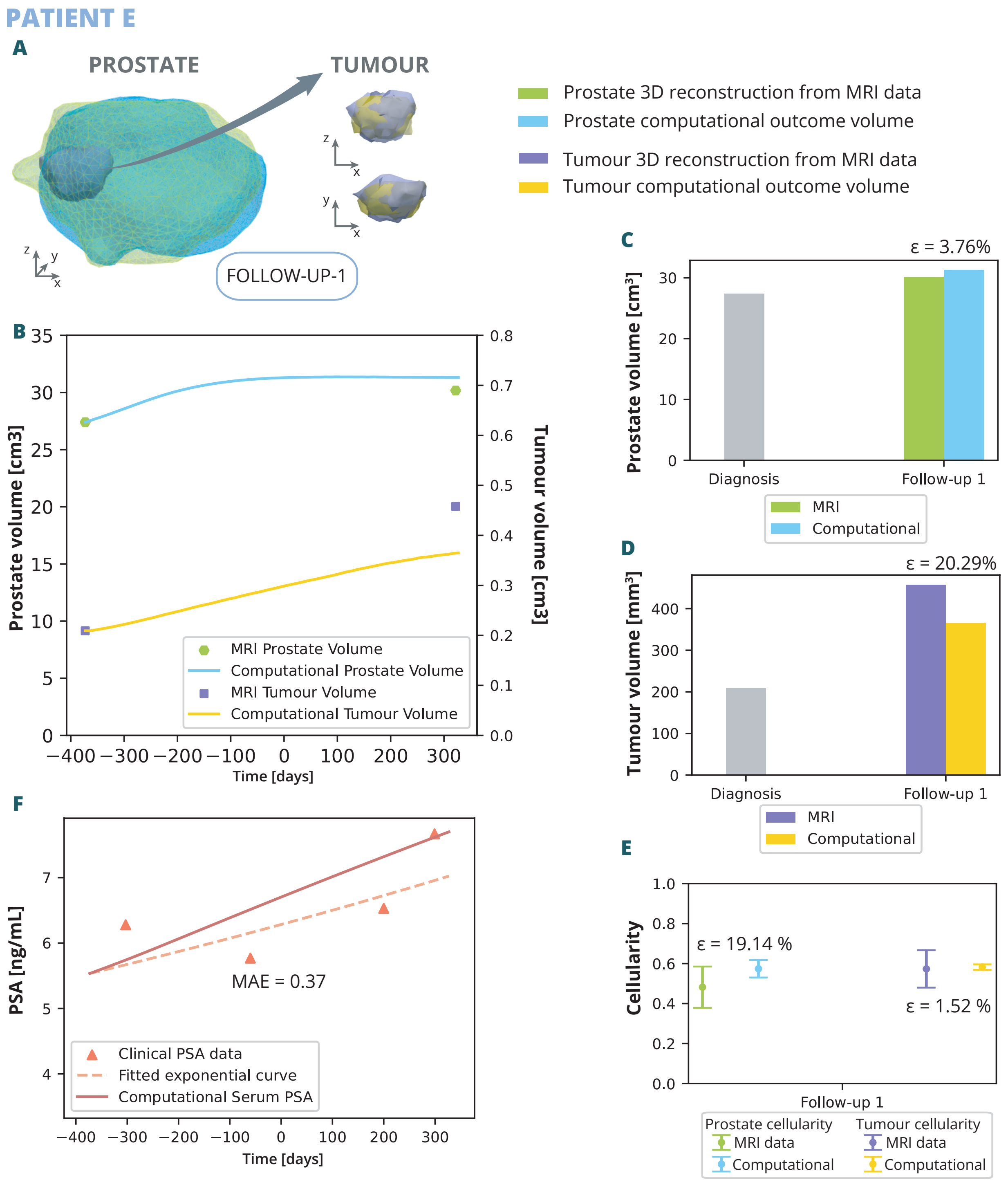

Figure S5. Patient E simulation results: (A) shows the simulated geometries of the prostate and the tumour compared to the MRI ones. In (B) the growth of the prostate and tumour volume are represented and compared to the MRI segmented volumes. In (C) and (D), these volumes have been represented with a bar chart for follow up 1 in order to make a clearer comparison, for the prostate and tumour, respectively. (E) represents the overall cellularity in the prostate observed in MRI as opposed to computational outcome. Beside, the cellularity in the tumour area is shown, also comparing the MRI data and computational outcome. Finally, in (F), the simulated serum PSA is compared to to the clinical observations.

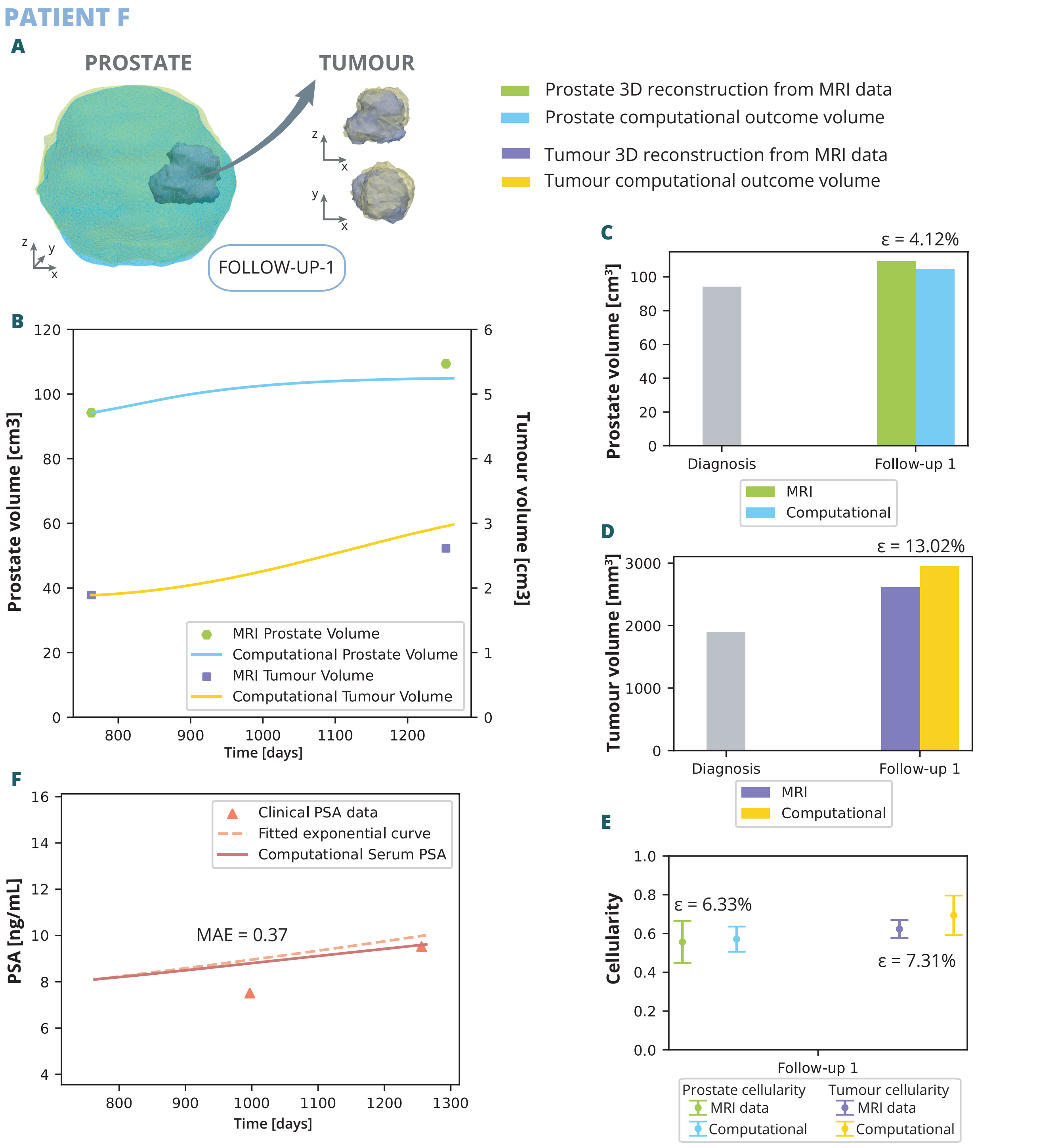

Figure S6. Patient F simulation results: (A) shows the simulated geometries of the prostate and the tumour compared to the MRI ones. In (B) the growth of the prostate and tumour volume are represented and compared to the MRI segmented volumes. In (C) and (D), these volumes have been represented with a bar chart for follow up 1 in order to make a clearer comparison, for the prostate and tumour, respectively. (E) represents the overall cellularity in the prostate observed in MRI as opposed to computational outcome. Beside, the cellularity in the tumour area is shown, also comparing the MRI data and computational outcome. Finally, in (F), the simulated serum PSA is compared to to the clinical observations.
